# The evolution of sex determination associated with a chromosomal inversion

**DOI:** 10.1038/s41467-018-08014-y

**Published:** 2019-01-11

**Authors:** Heini M. Natri, Juha Merilä, Takahito Shikano

**Affiliations:** 1 Ecological Genetics Research Unit, Organismal and Evolutionary Biology Research Programme, Faculty of Biological and Environmental Sciences, University of Helsinki, P.O. Box 65, FI-00014 Helsinki, Finland; 2 Institute for Research Initiatives, Nara Institute of Science and Technology, Ikoma, Nara, 630-0192 Japan

## Abstract

Sex determination is a fundamentally important and highly diversified biological process, yet the mechanisms behind the origin of this diversity are mostly unknown. Here we suggest that the evolution of sex determination systems can be driven by a chromosomal inversion. We show that an XY system evolved recently in particular nine-spined stickleback (*Pungitius pungitius*) populations, which arose from ancient hybridization between two divergent lineages. Our phylogenetic and genetic mapping analyses indicate that the XY system is formed in a large inversion that is associated with hybrid sterility between the divergent lineages. We suggest that a new male-determining gene evolved in the inversion in response to selection against impaired male fertility in a hybridized population. Given that inversions are often associated with hybrid incompatibility in animals and plants, they might frequently contribute to the diversification of sex determination systems.

## Abstract (web summary)

Turnovers in sex determination systems occur quite frequently, yet the evolutionary drivers of these turnovers are not well understood. Here, the authors study the sex determination systems in sticklebacks and propose chromosomal inversions as a possible driver of the evolution of sex determination.

## Introduction

Sex determination is a universal process in sexual organisms with a wide array of biological and evolutionary consequences and thus might be expected to be highly conserved during evolution as observed in a number of other basic biological processes. Yet, the mechanisms of sex determination are remarkably diverse, particularly in some taxa, such as fish, amphibians and reptiles, in which the genetic basis of sex determination can vary between closely related species or even within the same species^1–3^. This fact raises two important questions as to why the mechanisms of sex determination are so diverse and how transitions in sex determination systems occur. Extensive theoretical studies have proposed several hypothetical models for the evolution of sex determination systems, such as genetic drift^4^, pleiotropic benefits^4,5^, sex ratio selection^6–8^, and sexual antagonism^9–11^. Most of these models emphasize the importance of natural selection on fitness differences that are directly caused by, or indirectly associated with, a novel locus as a driving force promoting transitions in sex determination systems^12,13^. Despite the extensive theoretical work, possible empirical evidence is currently available only, to our knowledge, from a case study for the sexual antagonism hypothesis^14^, in which novel sex determiners evolve near sexually antagonistic genes by resolving intralocus sexual conflict^10,11^. Accordingly, the mechanisms that lead to transitions in sex determination systems remain mostly unknown. In particular, it is of fundamental importance to understand the factors that destabilize an existing sex determination system and drive the evolution of a new system.

The stickleback fish family (Gasterosteidae) exhibits a rapid turnover of sex determination and sex chromosome systems, and thus their sex chromosomes are considered to be young^15^. However, morphologically and genetically highly distinct X and Y chromosomes are found in the nine-spined stickleback (*Pungitius pungitius*)^15–17^. These chromosomes correspond to linkage group (LG) 12, which consists of chromosome (Chr) 12 and a part of Chr7 in the three-spined stickleback (*Gasterosteus aculeatus*)^18^, and the sex-determining gene is located in the *G. aculeatus* Chr12 region^18,19^, which is not involved in sex determination in *G. aculeatus*^20^ or in the brook (*Culaea inconstans*) or four-spined stickleback (*Apeltes quadracus*)^15^, phylogenetically close relatives of *Pungitius*^21^.

Here we demonstrate that the highly distinct X and Y chromosomes have formed recently in particular *P. pungitius* populations through hybridization between the ancestors of two distinct *Pungitius* lineages. We show that a large chromosomal region of the X and Y is derived from different lineages, where the orientation of the region is inverted from each other. The inversion region is associated with hybrid sterility between these lineages. Our results indicate that a new male-determining gene occurred on the inversion segment derived from the lineage that has a ZW sex determination system. We suggest that a new XY sex determination system has evolved in response to selection for hybrid incompatibility caused by the chromosomal inversion.

## Results

### Different sex determination systems

**Fig. 1:**
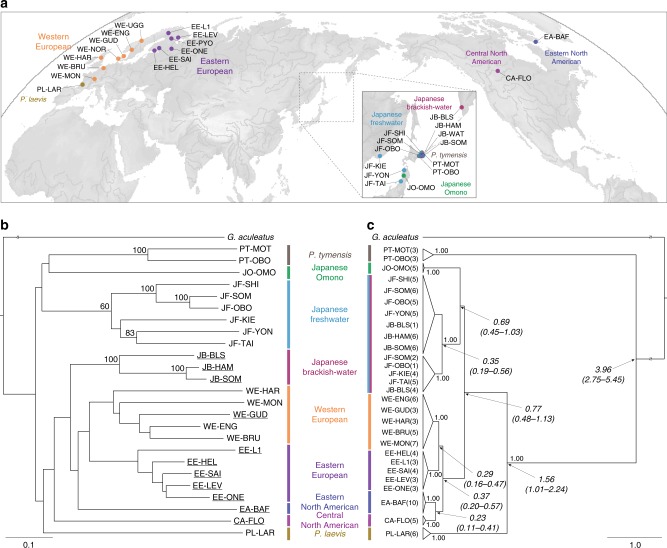
Phylogenetic relationships among *Pungitius* populations. **a** Sampling sites of *Pungitius* populations and distributions of different lineages used in this study. **b** Phylogenetic relationships among 25 populations based on 46 autosomal loci. Populations with an XY sex determination system are underlined. Bootstrap values (≥60) are shown at the nodes. **c** Mitochondrial phylogeny among 25 populations based on cytochrome *b* sequences. Posterior probabilities (≥0.95) are indicated at the nodes. The number of individuals observed in each clade is given in parentheses after the population code. Divergence time estimates (in millions of years) are shown in italics with the 95% highest posterior density interval in parentheses

Based on a global phylogenetic analysis of 25 *Pungitius* populations using 46 autosomal short tandem repeat (STR) loci and mitochondrial cytochrome *b* sequences, we identified nine highly divergent lineages, corresponding to *P. tymensis* (PT), Japanese Omono (JO), Japanese freshwater (JF), Japanese brackish-water (JB), western European (WE), eastern European (EE), eastern North American (EA), central North American (CA), and *P. laevis* (PL) lineages (Fig. 1, Supplementary Table 1, and Supplementary Data 1). According to a recent study on East Asian *Pungitius* fishes^22^, the JF and JO lineages correspond phylogenetically to *P. sinensis* and *P. kaibarae*, respectively, which are often taxonomically classified as subspecies of *P. pungitius*^23,24^, whereas all the remaining lineages except the PT and PL belong to *P. pungitius*. A single mitochondrial clade is found for the JF and JB lineages, possibly due to recent mitochondrial introgression from the former to the latter^25^. Based on the cytochrome *b* data, mitochondrial divergence between the PT and other lineages is indicated to have occurred around 4.0 million years ago, and the splits of the latter lineages are dated to approximately 1.56–0.23 million years ago (Fig. 1c). Incongruence between the nuclear and mitochondrial phylogenies is likely caused by the introgression of ancestral *P. pungitius* mitochondria into the JO and JF lineages^22^, which could have occurred prior to the initiation of their mitochondrial divergence around 0.77 million years ago (Fig. 1c). A survey with six loci located on *G. aculeatus* Chr12 indicates male-specific alleles at all or most of the loci in all JB, CA, EA, and EE populations, whereas other populations have no male-specific alleles, except for one WE population where genetic admixture occurs between the WE and EE lineages^26^ (Fig. 1b and Supplementary Table 2).

**Fig. 2:**
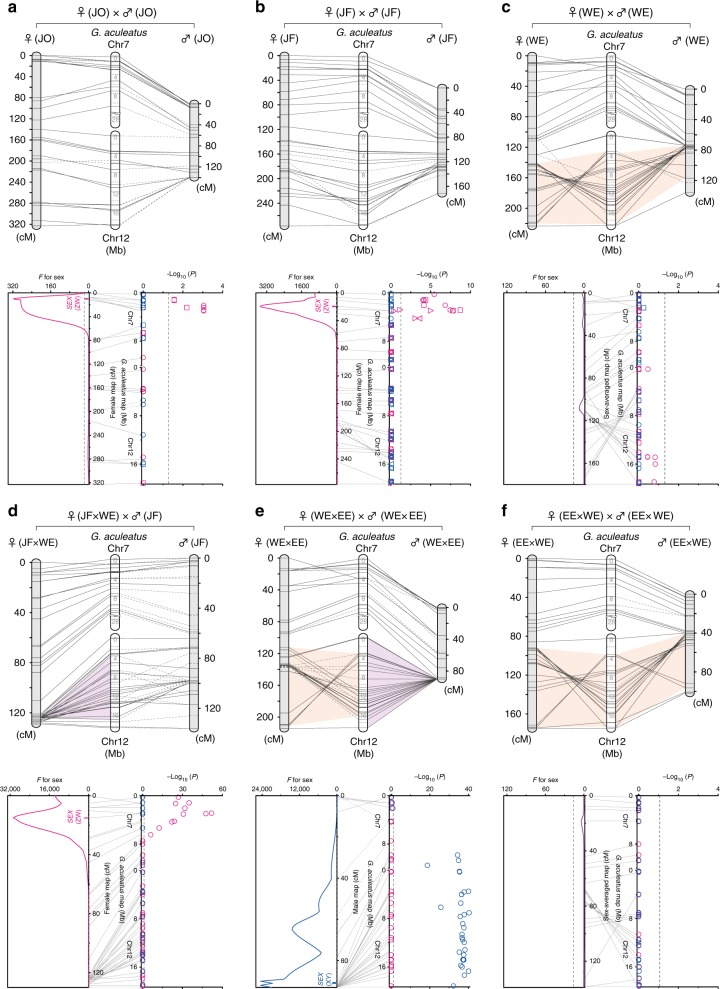
Comparative genetic maps and sex determination systems. Comparative genetic maps of *Pungitius* LG12 and *G. aculeatus* Chr7 and Chr12 in the JO lineage (**a**), the JF lineage (**b**), the WE lineage (**c**), a backcross between an F_1_ hybrid female (JF female × WE male) and a JF male (**d**), and F_2_ crosses between a WE female and an EE male (**e**) and between an EE female and a WE male (**f**). Female and male maps are shown to the left and right of *G. aculeatus* physical maps, respectively, on the top. Orange shaded areas indicate large inverted chromosomal regions between *Pungitius* and *G. aculeatus*, whereas purple shaded areas indicate non-recombining chromosomal regions. Dashed lines in comparative maps indicate uninformative (i.e., non-segregating) loci in the corresponding sex. QTL positions for sex and significance levels of associations between sex and loci are shown in the graphs to the left and right at the bottom, respectively. Dashed lines indicate statistically significant levels (*P* = 0.05). The means and 95% confidence intervals of QTL positions are indicated to the left of the map positions. In the graphs on the right, maternally and paternally segregating alleles are indicated in red and blue, respectively. Different symbols represent data from different families

To investigate the sex-determining region and possible chromosomal rearrangements in LG12, we performed genetic mapping in the JO, JF, and WE lineages, as well as three interlineage crosses including a backcross between the JF and WE lineages and reciprocal F_2_ crosses between the WE and EE lineages. In all the lineages and crosses, LG12 comprises the whole region corresponding to Chr12 and a part of Chr7 of the *G. aculeatus* genome (Fig. 2), as is found in the EE lineage^18^. However, while the marker orientation of the JO and JF lineages is mostly consistent with that of *G. aculeatus* (Fig. 2a, b), a large chromosomal region corresponding to 3.5–18.9 Mb (80.2%) of *G. aculeatus* Chr12 is inverted in the WE lineage (Fig. 2c). Accordingly, in the backcross of an F_1_ hybrid female (JF female × WE male) to a JF male, recombination is mostly suppressed in this region in the hybrid female meiosis, as expected in the presence of an inversion (Fig. 2d). Likewise, recombination is restricted across the region of *G. aculeatus* Chr12 in the male meiosis of the F_2_ cross between a WE female and an EE male (Fig. 2e). In contrast, such recombination suppression is not found in the female meiosis of this cross or in the female or male meiosis of the reciprocal F_2_ cross, where the orientation of the inversion region is congruent with that of the WE lineage (Fig. 2e, f).

Consistent with earlier studies of EE and CA populations^18,19^, phenotypic sex is perfectly associated with paternal alleles at loci of *G. aculeatus* Chr12 in the F_2_ cross between a WE female and an EE male, indicating male heterogametic (XY) sex determination (Fisher’s exact test, *P* = 4.4 × 10^−41^–1.8 × 10^−26^; Fig. 2e). The sex-determining gene is mapped to the region of suppressed recombination in the male map (*F* = 24519, *P* < 0.01; Fig. 2e). However, in the JO and JF lineages, a significant association with sex is found for maternal alleles at loci of *G. aculeatus* Chr7 (Fisher’s exact test, *P* = 9.2 × 10^−4^–3.0 × 10^−2^ and 1.9 × 10^−9^–8.7 × 10^−4^, respectively; Fig. 2a, b), indicating that these lineages have a female heterogametic (ZW) sex determination system. The sex-determining gene is mapped to a region of 10.0 cM in the female map (322.7 cM) of the JO lineage (*F* = 318, *P* < 0.01; Fig. 2a) and a region of 19.4 cM in that (276.6 cM) of the JF lineage (*F* = 3492, *P* < 0.01; Fig. 2b), which are distinct from the inversion region where the sex-determining gene of the XY system is located. Similar patterns of sex association are observed in the backcross progeny of the JF and WE lineages (Fisher’s exact test, *P* = 1.4 × 10^−52^–8.5 × 10^−7^; Fig. 2d), as well as in F_1_ hybrids between a JO female and a JF male (*P* = 2.4 × 10^−5^–1.5 × 10^−4^; Supplementary Fig. 1a). In contrast, in the WE lineage or in the F_2_ cross between an EE female and a WE male, no significant association with sex is found for any of the loci across LG12 (Fisher’s exact test, *P* = 0.15–1.00; Fig. 2c, f), where no significant quantitative trait locus (QTL) for sex is found (*F* = 7.2 or 4.2, respectively, *P* > 0.05; Fig. 2c, f), indicating that LG12 is not involved in sex determination in the WE lineage. In F_1_ hybrids between a JF female and a JB male, both of which are heterogametic (ZW and XY, respectively), sex is associated with maternal alleles at the loci where the sex-determining gene of the JF lineage is mapped (Fisher’s exact test, *P* = 9.7 × 10^−6^–9.3 × 10^−4^), whereas paternal alleles at loci on LG12 do not show any association with sex (*P* = 0.61–1.00; Supplementary Fig. 1b).

### Evolution of X and Y chromosomes

**Fig. 3:**
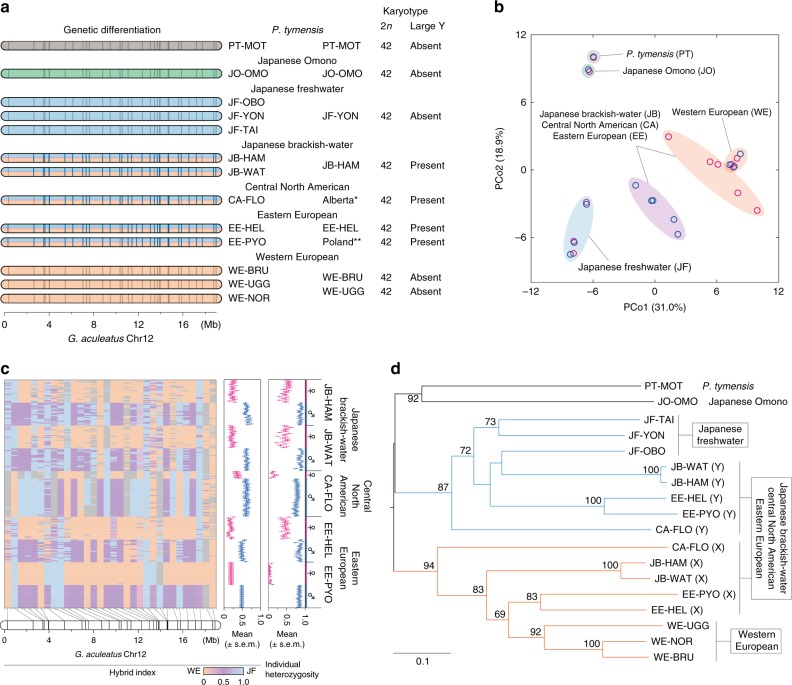
Genetic differentiation and evolutionary relationships of X and Y chromosomes. **a** Sex-specific genetic differentiation in the region of *G. aculeatus* Chr12 and the presence or absence of a large male-specific chromosome in 13 populations from seven lineages. Loci with male-specific alleles are indicated in blue. Locus positions are based on the *G. aculeatus* genome. Different chromosome colors represent different origins, as indicated by phylogenetic analyses based on *G. aculeatus* Chr12 loci (see **d**). Karyotype data for Alberta* and Poland** are based on Ross et al.^15^. and Ocalewicz et al.^16^, respectively. **b** Principal coordinate analysis among 13 populations. PCo1 and PCo2 values are plotted on the *x* and *y* axes, respectively. Females (red) and males (blue) are indicated separately. **c** The hybrid index and individual heterozygosity of each individual from five populations belonging to three lineages with an XY sex determination system. Hybrid index estimates are given for each locus. The hybrid index is expected to be 0 or 1 when a locus has two alleles unique to the WE or JF lineages, respectively, and 0.5 when a locus has two alleles, each of which is present in a different lineage. The hybrid index level for each locus is represented with a color gradient. Uninformative loci are shown in gray. Mean hybrid index values and individual heterozygosity are given for each individual. Bars represent standard error of the mean. Female and male individuals are shown in red and blue, respectively. **d** Phylogenetic relationships among 13 populations based on *G. aculeatus* Chr12 loci. X- and Y-chromosomal haplotype data are used for three lineages with an XY sex determination system. Bootstrap values (≥60) are given near the nodes. The tree is rooted at the midpoint

To genetically characterize the X and Y chromosomes of phylogenetically distinct lineages, we investigated sex-associated genetic variation across a region corresponding to *G. aculeatus* Chr12, where the sex-determining gene of the XY system is located, using 13 populations (Fig. 3a). All populations belonging to the JB, EE, and CA lineages exhibit male-specific alleles in 82.9–85.7% (2.6–19.2 Mb) of the 35 STR loci investigated, indicating high genetic divergence between the X and Y chromosomes (Fig. 3a and Supplementary Data 2). In contrast, none of the loci show sex-associated differentiation in the PT, JO, JF, or WE lineages. Earlier studies in EE and CA populations have shown that the Y chromosome is much larger than the X chromosome and autosomes^15,16^. Similarly, our cytogenetic analyses indicate a large male-specific chromosome in the JB and EE lineages, although no such chromosome is observed in the PT, OM, JF, or WE lineages (Fig. 3a and Supplementary Fig. 2). Thus the genetically and morphologically distinct X and Y chromosome pair is found in all the *P. pungitius* lineages except WE.

We further assessed the evolutionary origin and divergence of the X and Y chromosomes based on a phylogenetic framework with the 13 populations. Principal coordinate analysis indicates two distinct genetic clusters for females and males in the populations of the JB, CA, and EE lineages (Fig. 3b), although single clusters are found for the other lineages. PCo1 and PCo2 scores for the females of these populations are similar to those of the WE populations, whereas these scores for the males are intermediate between those of the WE and JF populations. Likewise, hybrid indices indicate that the females of these three lineages have alleles observed only in the WE populations at most loci, whereas the males have both WE- and JF-specific alleles at each of several loci (Fig. 3c). Accordingly, the males have higher heterozygosity than the females in the populations of the three lineages (Mann–Whitney *U* test, *P* < 0.001; Fig. 3c and Supplementary Data 2). Moreover, the phylogenetic tree demonstrates that the X and Y haplotypes of these populations cluster together with the WE and JF populations, respectively (Fig. 3d). These results indicate that the *G. aculeatus* Chr12 region of the X and Y chromosomes has the same origin as that of the WE and JF lineages, respectively. The inferred evolutionary origins of these chromosomes are consistent with the fact that recombination in the inversion region of LG12 is suppressed in the male meiosis of the lineages with the XY system^18,19^ as expected for the chromosome pair derived from both WE and JF lineages (Fig. 2b–d), as well as with the fact that the orientation of the inversion region in the X chromosome is identical to that in the WE lineage (Fig. 2e, f). The results might also imply two alternative scenarios for the evolutionary history of the XY system: first, the XY chromosome pair has been formed by hybridization between the ancestors of the WE and JF lineages; and second, while the XY system is ancestral in the *Pungitius* genus, the JF and WE lineages have lost the X and Y chromosomes, respectively, and acquired new sex determination systems. Nonetheless, since the JF and JO lineages have the same ZW system, it is evident that the ZW system has evolved prior to the divergence of these lineages. Moreover, while the JO and PT lineages belong phylogenetically to the Eastern clade that also includes the JF lineage^22^, the chromosomal region of the JO and PT lineages does not have genetic similarities to that of either the X or the Y chromosome (Fig. 3d; Supplementary Table 3). More specifically, the average genetic distances between the JF lineage and Y haplotypes (0.62) and between the WE lineage and X haplotypes (0.59) are equivalent to those within each lineage or haplotype group (0.31–0.61), whereas the genetic distances of the JO and PT lineages to the X and Y haplotypes are similarly high (0.90–0.93), corresponding to those between the JF and WE lineages (0.88) and between the X and Y haplotypes (0.92; Supplementary Table 3). These facts indicate that the XY chromosome pair is derived from hybridization between the ancestors of the WE and JF lineages.

### Hybrid incompatibility caused by an inversion

**Fig. 4:**
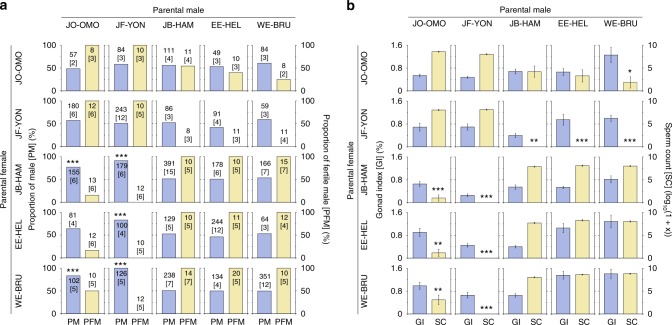
Hybrid incompatibility in interlineage crosses. **a** The proportions of adult males and of fertile males in five lineages and their crosses. Sterile and fertile males are identified based on the absence and presence of sperm in the testes, respectively. Sample numbers are shown in the graphs with family numbers in brackets. Significant deviations from a 1:1 sex ratio are indicated with asterisks (***P < 0.001). **b** Gonad index, as a measure of testis size, and sperm count in males in the same cross combinations as in **a**. Means and standard errors are shown. The data are based on the same individuals used for the analyses of sterility and fertility in **a**. Hybrids with significant differences from both parental populations are indicated with asterisks (*P < 0.05, **P < 0.01, and ***P < 0.001)

To address the possible evolutionary causes of the XY system, we investigated genetic incompatibilities in 20 crosses among five lineages (Fig. 4). While fertilization rate, hatching rate, and larval survival are similar to those in one or both of the parental populations in all the crosses (Supplementary Table 4), the sex ratio of the hybrids is biased toward males (76.8–100.0%) in crosses of JF males with JB, EE, and WE females and those of JO males with JB and WE females (binomial test, *P* = 5.9 × 10^−37^–3.6 × 10^−10^; Fig. 4a and Supplementary Table 5). In 4 out of the 5 crosses, survival to adult stage is <50% (Supplementary Table 5). However, despite a relatively high survival rate (69.6%) in the hybrids between JB females and JF males, most individuals (91.1%) are males, implying that male-biased sex ratios are unlikely to be caused by female-specific mortality. Moreover, while hybrid males in all crosses have similar testis sizes as one or both of the parental populations, no sperm is observed in the testes of hybrid males in reciprocal crosses of the JF with JB, EE, or WE lineages (Fig. 4a, b). Likewise, a fraction of hybrid males (45.5–84.6%) do not have sperm in reciprocal crosses of the JO with JB, EE, or WE lineages. Similar patterns of hybrid male sterility are observed in backcrosses with the parental populations (Supplementary Table 6). In all the backcrosses, fertilization rates of eggs produced by gravid hybrid females, as well as hatching rate and larval survival of fertilized eggs, are similar to those in one or both of the parental populations (Supplementary Table 6). These results indicate that sex ratio bias and intrinsic male sterility occur in hybrids of particular lineage combinations, including those of the JF and WE lineages, and that male hybrids are fertile when the lineages with the XY system are crossed to the WE lineage but sterile when they are crossed to the JF lineage.

**Fig. 5:**
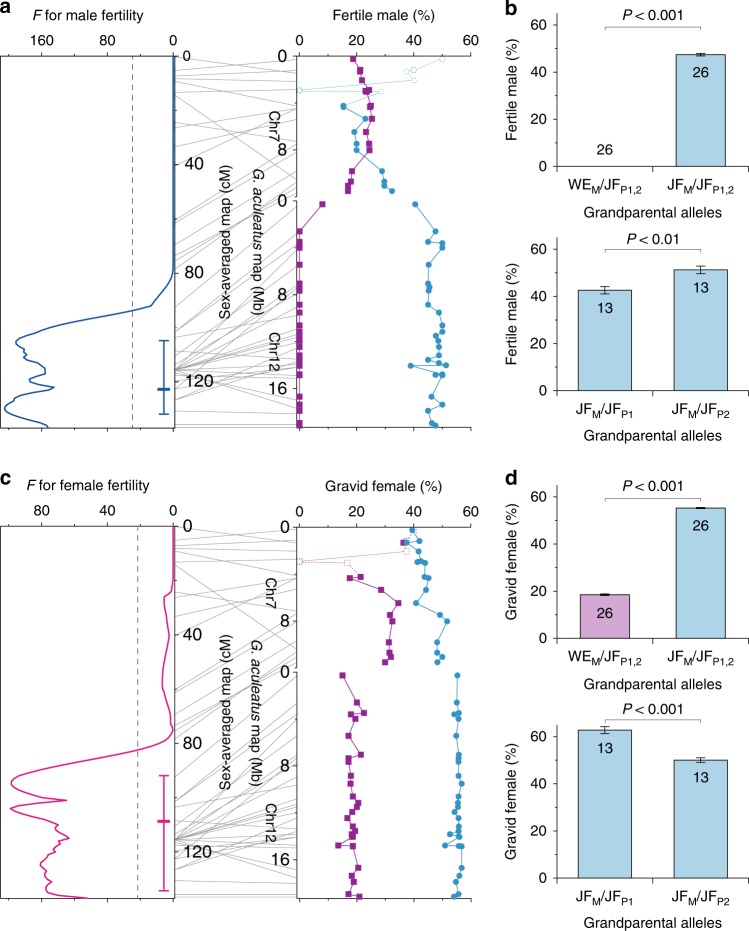
QTL region for hybrid male and female fertility. **a** QTL analyses for male fertility (left) and the proportions of fertile males with different allelic combinations at each locus (right) in the backcross. **b** The proportions of fertile males with different allelic combinations in the inversion region (3.5–18.9 Mb of *G. aculeatus* Chr12). **c** QTL analyses for female fertility (left) and the proportions of gravid females with different allelic combinations at each locus (right) in the backcross. **d** The proportions of gravid females with different allelic combinations in the inversion region. In **a**, **c**, dashed lines in the graphs on the left represent statistically significant levels (*P* = 0.05) of the *F*-value. The mean and 95% confidence interval of a QTL position are indicated to the left of the map positions. In the graphs on the right, data for individuals with both WE and JF alleles at each locus are indicated in purple and those with only JF alleles are indicated in cyan. Open symbols represent data from ≤10 individuals due to the presence of sex-linked alleles. In **b**, **d**, means and standard errors for loci with grandparental WE and JF alleles (WE_M_/JF_P1,2_) and those with two grandparental JF alleles (JF_M_/JF_P1,2_) are shown on the top. Means and standard errors for loci with two JF alleles, one from the maternal grandmother (JF_M_) and the other from the paternal grandmother (JF_P1_) or grandfather (JF_P2_), are shown at the bottom. Data for individuals with both WE and JF alleles are indicated in purple and those with only JF alleles are indicated in cyan. Numbers of loci analyzed are indicated in the graphs

To assess whether the large inversion in LG12 is involved in hybrid fertility, we performed genetic mapping of fertility in both sexes using the backcross between the JF and WE lineages with 49 polymorphic loci distributed across LG12. Both fertile and sterile males are found in the backcross progeny, including 23.3% (21/90) of fertile individuals with sperm in the testes. A significant QTL for male fertility is detected in the region of 123.2 cM (95% confidence interval, 104.0–131.0 cM) in the sex-averaged map (*F* = 205.2, *P* < 0.01; Fig. 5a), where the large inversion is located. While all individuals heterozygous for both grandparental WE and JF alleles in this region are sterile, 47.4% of individuals homozygous for JF alleles are fertile (Mann–Whitney *U* test, *P* < 0.001; Fig. 5b). In the latter group, the proportion of male fertility differs slightly but significantly between individuals with alleles from the different paternal grandparents in this region, showing a lower proportion of fertility in individuals with alleles from the paternal grandmother (42.6%) than those with alleles from the paternal grandfather (51.3%; Mann–Whitney *U* test, *P* = 0.002; Fig. 5b). In the females of the backcross generation, the proportion of gravid individuals is 40.5% (45/111). A significant QTL for female fertility, as measured by gravidity status (with vs. without eggs), is found in the region of 108.4 cM (95% confidence interval, 92.0–135.0 cM) in the sex-averaged map (*F* = 99.1, *P* < 0.01; Fig. 5c), which corresponds to the QTL region of male fertility. The proportion of gravid females is 18.5% in individuals heterozygous for both grandparental WE and JF alleles in the inversion region, whereas it is 55.3% in individuals homozygous for JF alleles (Mann–Whitney *U* test, *P* < 0.001; Fig. 5d). In the latter group, individuals with alleles from the paternal grandmother show a higher proportion of gravid females (62.8%) than those with alleles from the paternal grandfather (50.0%; Mann–Whitney *U* test, *P* < 0.001; Fig. 5d). Therefore, while both male and female fertility is drastically lowered when the inversion region is heterozygous, this region also affects male and female fertility with sexually antagonistic effects when it is homozygous.

## Discussion

**Fig. 6:**
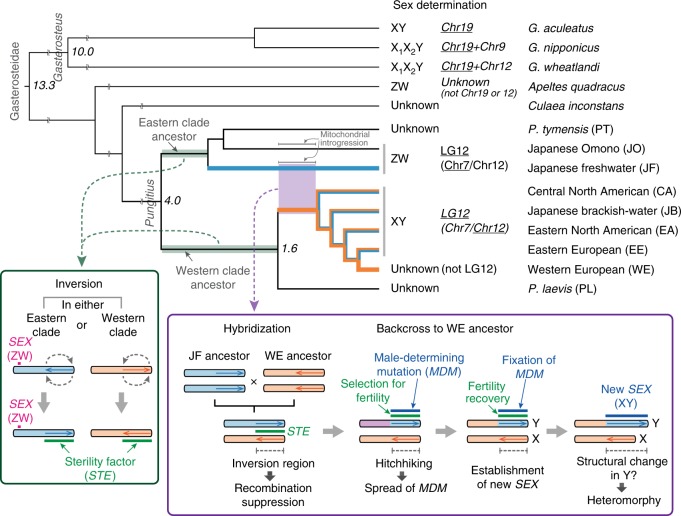
Evolution of sex determination in Gasterosteidae fishes. Hypothetical processes involved in the evolution of the XY sex determination system in the *Pungitius* genus are shown in the boxes. The green shading on the Eastern and Western clades indicates the evolution of the inversion and sterility factor. The blue and orange colors in the phylogenetic tree represent different chromosomal origins of the inversion region. The branch leading to the *P. pungitius* lineages in the Western clade is indicated in orange to represent the ancestral condition prior to the hybridization event shown in the purple box. Phylogeny and sex determination are based on this and earlier studies^15, 21, 22, 47, 70^. Sex-determining chromosomes are underlined, and heteromorphic sex chromosomes are indicated in italic. Divergence times based on fossil records^70^ and mitochondrial divergence are shown in italics (in millions of years)

Our study revealed that both ZW and XY sex determination systems exist in the *Pungitius* lineages. While the *Pungitius* lineages are classified phylogenetically into two major clades: the Eastern clade, comprising the JF, JO, and PT lineages, and the Western clade, including the PL and all *P. pungitius* lineages, the highly distinct XY chromosome pair is found only in particular *P. pungitius* lineages in the Western clade. Our results indicate that the XY chromosome pair has arisen through hybridization between the ancestors of the WE and JF lineages, suggesting that the male-determining gene of the XY system has evolved on a large non-recombining chromosome segment derived from the JF lineage, which has a ZW locus in a different chromosomal position where recombination occurs frequently. In the comparative mapping of the JF and WE lineages, a large inversion is found in the region corresponding to the sex-determining region of the XY system, thereby causing recombination suppression in heterozygotes. Thus it appears that recombination in the sex-determining region of the XY system became suppressed owing to the inversion when the chromosome pair was formed. Since the orientation of the inversion region is consistent between the JF and JO lineages and between the WE lineage and the X chromosome of the EE lineage, the inversion likely occurred in either the ancestor of the Eastern clade or that of the Western clade after the divergence of these two clades (Fig. 6). Under the assumption that the ancestor of *Pungitius* fishes had the same chromosomal orientation as *G. aculeatus*, the inversion would most parsimoniously have occurred in the ancestor of the Western clade.

It is noteworthy that the inversion region is associated with hybrid sterility. Inversions are thought to accumulate positively selected alleles that are beneficial to a population while these alleles are conversely often genetically incompatible between populations^27^. Thus inversions are often associated with genetic incompatibilities between populations in which inverted and non-inverted chromosomal segments are alternatively fixed^27,28^. The inversion-associated hybrid sterility observed in our study could have evolved under two plausible alternative scenarios. First, it might have arisen prior to hybridization between the ancestors of the JF and WE lineages after the Eastern and Western clades had diverged from each other. Second, it might have occurred as a result of the evolution of the XY system. Our results show that hybrid sterility occurs in all combinations between the populations of the Eastern and Western clades but not within them, irrespective of the presence or absence of the XY chromosomes. In addition, no hybrid sterility is found between *P. pungitius* and *P. platygaster*^29^, despite their ancient divergence (2.3 million years ago) within the Western clade^30^. Likewise, hybridization and introgression occur between *P. pungitius* and *P. laevis* in sympatry^31^. Thus it is more plausible that genetic factors underlying hybrid sterility arose in the inversion region in one of the two clades prior to hybridization between the ancestors of the JF and WE lineages (Fig. 6). Our results also show that the adult sex ratio is biased toward males in most crosses between females of Western clade populations and males of Eastern clade populations but not in the reciprocal crosses, as previously found in crosses between homogametic females (XX) and males (ZZ) in other organisms^32,33^. Our study demonstrates that, in hybrids between a JF female (ZW) and a JB male (XY), sex is determined by the genotype of the ZW system without any impact of the male-determining gene of the XY system, suggesting that the Z allele of the ZW system has a male-determining function in the absence of the dominant female-determining W allele. Therefore, the male-biased sex ratios observed in our study likely occur due to the action of the male-determining allele of the ZW system, independently of the XY system. The result also implies that the sex ratio distortion is not associated with the inversion region where the sex-determining gene of the XY system is located. In addition, it seems unlikely that the male-biased sex ratio distortion can be the primary cause of the evolution of the new male-determining gene of the XY system, whose action is suppressed in the presence of the ZW system. By contrast, the fact that the XY system is found in the inversion region that is associated with hybrid sterility is in line with the theoretical expectation that a new sex-determining gene can evolve in response to fitness differences caused by genetic variation that is in strong linkage disequilibrium with it^12,13^.

It is of interest to consider how the ancestral population of the XY lineages could have gained fertility and why the XY system could have formed in the inversion region, which should be key to the evolution of the new sex determination system. Owing to impaired fertility and biased sex ratio, hybrid offspring would have experienced a dramatic plunge in reproductive success following hybridization between the ancestral populations of the WE and JF lineages. Given that fertility in the backcross individuals is affected by allelic combinations in the inversion region even when it is homozygous (i.e., JF_M_/JF_P1_ vs. JF_M_/JF_P2_ in Fig. 5b, d), hybrid sterility is likely caused by genes within this region rather than the structural consequences of the inversion per se as demonstrated in other organisms^34,35^. Thus it is possible that the genetic factors involved in hybrid sterility had not yet been completely fixed differently in the ancestral populations of the WE and JF lineages when hybridization occurred. In such a situation, at least a portion of hybrid males could have been fertile, as observed in the hybrids of the JO with WE and XY lineages, allowing the ancestral population of the XY lineages to become fully fertile through natural selection on standing genetic variation in the inversion region. As a consequence, it is likely that the current Y chromosome of the XY lineages became genetically compatible with the homologous chromosome of the WE lineage but lost compatibility with that of the JF lineage as indicated by impaired fertility when they are paired.

A new sex-determining mutation might have arisen in the inversion region of the chromosome derived from the JF lineage simply by chance, as generally hypothesized^36,37^. Yet, owing to recombination suppression in the region, selection for fertility of hybrid progeny would have rapidly changed the frequency of the mutant allele in a population as a result of genetic hitchhiking (Fig. 6). Our results show that the genetic impact of the inversion region on hybrid sterility is particularly severe for hybrid males. However, since females have a pair of X chromosomes originating from the ancestor of the WE lineage, with mitochondria phylogenetically close to this lineage in all the XY lineages except JB, which has recently experienced mitochondrial introgression^25^ (Fig. 1c), it appears that repeated backcrossing of hybrid male progeny to females of the WE ancestor occurred during the formation of the ancestral population of the XY lineages, which would also eliminate the impact of the sex-determining locus of the JF lineage from the population (Fig. 6). Therefore, hybrid male progeny were likely subject to strong selection to restore fertility. As theoretically expected, selection for hybrid male fertility would create strong linkage disequilibrium in the inversion region and spread the new male-determining gene in the ancestral population of the XY lineages^4,13,38^ (Fig. 6). It is also noteworthy that the inversion region is associated with sexually antagonistic effects on fertility in certain genetic backgrounds, suggesting that the fertility of hybrid progeny could be enhanced by sexually antagonistic selection favoring alternative alleles in females and males, which is again expected to spread the new male-determining gene in the population^9–11^. Regardless of whether selection to restore diminished fertility of the hybrid male progeny has acted in a male-specific or sexually antagonistic manner, the coincidence of male determination and hybrid male fertility due to strong linkage disequilibrium in the inversion region could have facilitated the establishment of a new sex determination system, consistent with the evolution of the new male-determining gene on the JF-derived chromosome that is now genetically compatible with the WE-derived homologous chromosome. The XY system is currently found in different *P. pungitius* lineages widely distributed in the Northern Hemisphere. While our data indicate that the origins of the X and Y chromosomes are common in these lineages, they cannot distinguish whether hybridization occurred once or multiple times. Likewise, given that the diversification and colonization processes of *Pungitius* fishes are largely unknown^22^, it is difficult to speculate on how the XY system has become widespread and prevalent in divergent lineages. However, since the XY lineages are commonly found in northern areas that have been strongly affected by historical glaciation events, the colonization of these areas might have taken place by different lineages that obtained the XY system before their divergence and migrated to different areas experiencing varying levels of gene flow from ancestors of other lineages. It is also possible that the XY system has spread to different lineages due to the introgression of the Y segment containing the male-determining gene from other lineages with the XY system through ancient gene flow.

Given that interspecific hybridization occurs in at least 10% of animal species and 25% of plant species^39^, hybrid incompatibility accompanied by inversions, which is often found between and within species^27,28^, might be rather commonly involved in the evolution of sex determination in various organisms. Furthermore, while it is questioned whether inversions lead to or result from sex chromosome differentiation^37^, our results hint that inversions can induce rapid evolution of heteromorphic sex chromosomes through their effects on recombination suppression (Fig. 6). Thus our study suggests not only the possible role of inversions in the evolution of sex determination systems but also their active role in sex chromosome differentiation, implying far greater evolutionary significance for inversions than is currently appreciated.

## Methods

### Samples

Several phylogenetically distinct lineages of *Pungitius* fishes have been identified in East Asia^25^, Europe^40^, and North America^41^. In total, 29 populations from these areas were used in this study (Supplementary Table 1). The fish were collected using hand nets, minnow traps, or seine nets. These populations were phylogenetically classified into nine lineages based on the present study as well as earlier studies^25,40,41^ (Supplementary Table 1). Three-spined sticklebacks were collected from the Baltic Sea (60° 12′ N, 25° 11′ E) and used as an outgroup in phylogenetic analyses. All samples were obtained in accordance with national and institutional ethical regulations with permission from the Finnish Food Safety Authority (#2736/425/2007, #1181/0527/2011, and #3701/0460/2011). All animal experiments were conducted under license from the Finnish National Animal Experiment Board (#STH379A and #PH1236A).

### Phylogenetic analyses

Total genomic DNA was extracted from ethanol-preserved fin clips using a silica-based purification^42^ or a phenol–chloroform extraction method^43^. Phylogenetic analyses were conducted for 25 globally distributed *Pungitius* populations based on 46 autosomal STR loci and mitochondrial cytochrome *b* sequences (Supplementary Table 2 and Supplementary Data 1). The phylogenetic lineages of the remaining populations were determined based on cytochrome *b* sequences (Supplementary Table 2). In total, 772 individuals (8–49 per population; Supplementary Table 2) were genotyped with the STR primer sets^17,44–46^ (Supplementary Data 1) using the Multiplex PCR Kit (Qiagen) in a reaction volume of 10 μl containing approximately 20 ng of template DNA, 1× Multiplex PCR Master Mix, 0.5× Q-Solution, and 2 pmol of each primer^47^. The reactions were performed as following: an initial denaturation step at 95 °C for 15 min, followed by 30 s at 94 °C, 90 s at 53 °C, and 60 s at 72 °C for 30 cycles with a final extension at 60 °C for 5 min. Alleles were analyzed using a MegaBACE 1000 automated sequencer (Amersham Biosciences) with ET-ROX 550 size standard (Amersham Biosciences) and scored using Fragment Profiler 1.2 (Amersham Biosciences). The number of polymorphic loci, expected heterozygosity, and *F*_IS_ were calculated using FSTAT 2.9.3^48^. Significant *F*_IS_ (10,000 permutations) was observed in only a few loci and populations (Gac4174 in JB-HAM and JB-SOM, Stn130 in JB-HAM and WE-BRU, Pprm7 in JF-OBO and JF-TAI, and Umf66 in WE-ENG; *P* < 0.05 with Bonferroni adjustment). The neighbor-joining (NJ) tree was constructed based on *D*_A_ distances^49^ with 1000 bootstrap replicates across loci using Populations 1.2.31^50^. The mitochondrial phylogeny was investigated based on an 1104-bp region of cytochrome *b*, which corresponds to 97% of the gene, with 149 individuals (3–10 per population; Supplementary Table 2). In this study, 43 individuals (4 from JF-KIE, 5 from JB-BLS, 8 from JB-WAT, 5 from NA-FLO, 1 from EE-PYO, 1 from EE-HEL, 8 from WE-UGG, 6 from WE-NOR, and 5 from WE-BRU) were sequenced using two primer sets with the BigDye Terminator v1.1 Cycle Sequencing Kit (Applied Biosystems)^40^, and the sequences of the remaining individuals were acquired from earlier studies^30,40^. Novel haplotype sequences were deposited in GenBank (accession nos. KR779233–KR779244, KY819041–KY819043). Phylogenetic relationships, as well as divergence times, were inferred using the Bayesian Markov chain Monte Carlo (MCMC) method implemented in BEAST 1.8.2^51^. For divergence time estimation, a cytochrome *b* sequence of *G. aculeatus* (GenBank accession no. KJ628012), which diverged from *Pungitius* approximately 13.3 million years ago^52^, was included in the analyses. The sequence data were partitioned by codon positions, and the nucleotide substitution model selected by jModelTest 2.1.7^53^ was applied to each position (K80+I, HKY, and HKY+G for the first, second, and third codon positions, respectively). The *BEAST analysis^54^ was performed using a Yule speciation process between species (i.e., *G. aculeatus*, PT, PL, and other lineages) and a coalescent process within species as the tree prior under a piecewise linear and constant root population size model and a strict clock model. To calibrate divergence ages, a lognormal prior distribution with a mean of 13.3 million years and standard deviation of 0.05 (95% quantiles = 12.0–14.7) was set for the node of *G. aculeatus* and *Pungitius*. The MCMC was run for 100,000,000 steps, with a burn-in of 20,000,000. Convergence of the MCMC run was inspected using Tracer 1.6 (http://tree.bio.ed.ac.uk/software/tracer/). Phylogenetic trees were visualized using FigTree 1.4.2 (http://tree.bio.ed.ac.uk/software/figtree/).

### Hybrid incompatibility

Hybrid incompatibility was investigated in 20 crosses among 5 (JO, JF, JB, EE, and WE) lineages based on multiple facets of post-zygotic isolation, including fertilization rate, hatching success, larval survival, adult sex ratio, and fertility. Adult or juvenile fish were obtained from JO-OMO, JF-YON, JB-HAM, EE-HEL, and WE-BRU and maintained in 90-l tanks at 16 °C in a laboratory. Interpopulation hybrid and intrapopulation control crosses were conducted with artificial fertilization, resulting in a total of 171 full-sib families (3–15 per cross; Supplementary Table 4). Crosses were performed using wild-caught fish or F_1_ progeny of the parental populations. Artificial fertilization was performed in a 15-ml Petri dish by mixing eggs squeezed from a gravid female and a sperm suspension prepared from testes macerated in Ringer’s solution^55^. After insemination, eggs were maintained in 15-ml Petri dishes (up to 30 eggs per dish) at 16 °C. The fertilization rate was examined 4–5 h after insemination (4- or 8-cell stage) under a dissecting microscope. Fertilized eggs were maintained in Petri dishes to measure the hatching rate, as well as the survival rate of larvae over a period of 7 days post-hatching. From 3 days post-hatching, larvae were fed twice daily with newly hatched brine shrimp (*Artemia* sp.) nauplii. After the measurements, the fish were reared in 2.8-l tanks at 16 °C for approximately 2 months and thereafter in 28-l tanks at 16 °C for ≥6 months to test their fertility. The hatching and larval survival rates were calculated based on the numbers of fertilized eggs and hatched eggs, respectively. Statistical significance between F_1_ crosses and the parental populations was determined using Mann–Whitney *U* test with Bonferroni correction.

Hybrid male fertility was examined using mature males displaying nuptial coloration based on testis size and sperm count. After the fish were anesthetized with Tricaine methanesulfonate (MS-222), the body weight was measured to the nearest 0.01 g, and the testes were removed immediately and weighed to the nearest 0.001 g. For each individual, the testes were cut into small pieces in 100 µl of deionized water, and the presence or absence of sperm was determined by observing the sperm suspension under a light microscope. Each positive sample was further diluted up to 1500 µl with deionized water, and sperm concentration was measured using a hemocytometer (Bürker chamber) to estimate total sperm count. Body weight, testis weight, and sperm count were measured twice for each individual, and the averaged values were used for analyses. Gonad index was used as an index of testis size, which is the ratio of testis weight to body weight, expressed as a percentage^56^. Sperm counts were transformed using the formula log_10_(1 + *x*). Statistical significance between F_1_ hybrids and the parental populations was evaluated using Mann–Whitney *U* test with Bonferroni correction. The sex ratio was also examined using adult individuals (about >4 months of age). Phenotypic sex was identified by visual inspection of secondary sexual characters (body and pelvic spine coloration) and/or gonads. Deviations from a 1:1 sex ratio were examined using a binominal test with Bonferroni correction. In addition, the fertility of F_1_ hybrids and the viability of their offspring were evaluated based on artificial crosses. The F_1_ hybrid males and females were backcrossed with females and males of the parental populations, respectively, using wild-caught individuals or outbred F_1_ or F_2_ progeny. Fertilization rate, hatching rate, and larval survival rate were investigated in the offspring as described above. Statistical significance between backcross progeny and the parental populations was assessed using Mann–Whitney *U* test with Bonferroni correction.

To enhance maturation, all experimental fish were reared under a 24-h photoperiod and fed twice a day with live brine shrimp (*Artemia* sp.) nauplii from 3 days post-hatching and frozen bloodworms (Chironomidae larvae) from approximately 8 weeks post-hatching.

### Karyotyping

Karyotype analyses were conducted for the PT, JO, JF, JB, EE, and WE lineages. Metaphase spreads were prepared from primary epithelial cells using the wild-caught fish or laboratory-bred F_1_ progeny of PT-MOT, JO-OMO, JF-YON, JB-HAM, EE-HEL, WE-BRU, and WE-UGG (minimum 3 males and 3 females per population). The fish were injected intraperitoneally with 0.5% colchicine solution at a dose of 2 μl/g of body weight and maintained in an aquarium for 4 h. After the fish were anesthetized with MS-222, the gill and spleen tissues were minced and incubated in 0.075 M KCl solution for 30 min on ice. Cells were fixed with Carnoy’s solution (methanol:acetic acid = 3:1), and cell suspensions were placed on glass slides and stained with 5% Giemsa solution.

### X and Y chromosome differentiation

Sex-associated genetic variation was investigated in the region corresponding to *G. aculeatus* Chr12 where the sex-determining gene of the XY system is located^17–19^ using 13 populations, including one PT, one JO, three JF, two JB, one CA, two EE, and three WE (Supplementary Data 2). In total, 613 individuals (35–52 per population; Supplementary Data 2) were genotyped with 35 STR loci located on *G. aculeatus* Chr12^17,19,45,57,58^ (Supplementary Data 1) using the Multiplex PCR Kit under the same conditions as described above. Of the 35 primer sets, 10 were developed in the present study following the procedures outlined in an earlier study^17^ and deposited in the National Center for Biotechnology Information (NCBI) Probe Database (Supplementary Data 1). The phenotypic sex of each individual was identified by secondary sexual characters and/or gonad inspection. Sex-associated genetic differentiation was assessed based on *F*_ST_ between sexes and sex-specific alleles^17,47^. *F*_ST_ values and their significance were evaluated using Genepop 4.2.1^59^ with 10,000 dememorization steps, 1000 batches, and 10,000 iterations per batch. In addition to the 13 populations, the presence or absence of male-specific alleles was examined in another 16 populations, which were also included in the phylogenetic analyses, using six loci (Ppsm11, Umf5, Ppsm3, Ppsm5, Ppam6, and Stn19; Supplementary Data 1) where Y-specific alleles are found in lineages with male heterogametic sex determination (Supplementary Data 2).

Genetic relationships among the females and males of the 13 populations were examined based on a principal coordinate analysis (PCoA) with the 35 STR loci using GenAlEx 6.5^60^. The analysis was conducted using the first and second principal coordinate axes, which accounted for 31.0% and 18.9% of the total genetic variation, respectively. Since the PCoA indicated a possible genetic admixture of two different lineages in the males, but not in the females, in the JB, CA, and EE lineages where genetically distinct X and Y chromosomes were identified (Supplementary Data 2), we conducted additional analyses to specifically address this issue. First, the hybrid index^61^ was estimated for each individual at the respective loci in the five populations of these lineages using GenoDive 2.0^62^. In this analysis, JF and WE populations were assumed to be the parental groups based on the results of the PCoA. Accordingly, the hybrid index is expected to be 0.5 for a locus with two alleles, each of which is derived from a different parental group, and 0 or 1 for a locus with two alleles unique to the WE or JF lineage, respectively. In addition, individual heterozygosity was compared between the females and males in each population. Second, phylogenetic relationships were assessed based on X- and Y-chromosomal alleles in the five populations of the three lineages (Supplementary Data 2). X- and Y-chromosomal haplotypes were estimated using the males of these populations with the ELB algorithm implemented in Arlequin 3.5.1^63^. Parameters were set as follows: alpha value = 0.02, epsilon value = 0.30, and gamma value = 0.02. This setting provided X- and Y-chromosomal haplotypes in accordance with the X- and Y-specific alleles identified throughout the *G. aculeatus* Chr12 region in each population. Phylogenetic relationships were assessed using all 13 populations to address the hypothesis that the genetically distinct X and Y chromosomes of these lineages originated from the WE and JF lineages, respectively. The NJ tree was constructed based on *D*_A_ distances with 1000 bootstrap replicates across loci using Populations 1.2.31.

### Comparative genetic mapping

Possible chromosomal rearrangements were examined in LG12 based on comparative mapping between the JF and WE lineages. Four and two outbred full-sib families were produced by artificial fertilization with F_1_ (3 pairs) or F_2_ progeny (1 pair) of JF-YON and wild-caught individuals of WE-BRU, respectively. For genetic mapping of JF-YON and WE-BRU, 142 (25–47 per family) and 144 individuals (72 per family) were used, respectively. Genetic mapping was also conducted for the JO lineage. Two full-sib families (31 and 24 individuals) were produced using wild-caught individuals of JO-OMO. As the numbers of available samples and polymorphic loci were relatively low in this lineage, the mapping analysis was performed by incorporating the information of maternally segregating loci in hybrids (34 individuals) between a JO-OMO female and a JF-YON male, each of which was produced by wild-caught individuals of the respective populations. To eliminate the effect of the JF lineage on the analysis, all paternal alleles were treated as monomorphic. All the fish were reared in 28-l tanks at 16 °C for at least 15 weeks after hatching.

In addition, we empirically tested whether recombination is suppressed in an inverted chromosomal region of the JF and WE lineages using their hybrids. The F_1_ hybrids were produced by artificial fertilization using a female from JF-YON and a male from WE-BRU and reared in a 28-l tank at 16 °C. Since the F_1_ hybrid males are sterile, a backcross was conducted using a female individual. One female of the F_1_ hybrids was naturally mated with one outbred F_2_ male of JF-YON in a 6-l tank in a zebrafish rack system (Aquaneering Inc.) to obtain fertilized clutches repeatedly. The backcross progeny were reared in 28-l tanks (20–30 fish per tank) at 16 °C for 40 weeks after hatching. In total, 217 individuals from 6 clutches were used for the analyses. We also investigated recombination patterns in reciprocal hybrids between the WE and EE lineages. Two F_1_ hybrid families were reciprocally produced by artificial fertilization with one female and one male from each of WE-BRU and EE-HEL and reared in 28-l tanks at 16 °C. To obtain F_2_ progeny, one female and one male in each F_1_ hybrid family were mated naturally in 6-l tanks in a zebrafish rack system. The F_2_ individuals of each reciprocal cross were reared in a 300-l tank at 15 °C for at least 34 weeks after hatching. The genetic analyses were performed using 144 individuals from the cross between a WE female and an EE male and 139 individuals from the reciprocal cross.

A total of 60 STR loci, 35 of which are located on Chr12 and 25 on Chr7 in the *G. aculeatus* genome (Supplementary Data 1), were genotyped for the individuals and parents of the mapping families, as well as the grandparents of the backcross family, using the Multiplex PCR Kit under the same conditions as described above. Alleles were analyzed using an ABI 3730 sequencer (Applied Biosystems) with the GeneScan 500 ROX size standard (Applied Biosystems) and scored using GeneMapper 5.0 (Applied Biosystems). The 17 novel primer sets developed for the *G. aculeatus* Chr7 region were deposited in the NCBI Probe Database (Supplementary Data 1). No segregation distortion was observed for any of the loci or families (chi-square test, *P* > 0.05 with Bonferroni adjustment), except for Ppsm15 in the F_2_ cross between an EE female and a WE male (*P* = 0.043). Genetic linkage maps were constructed with CRI-MAP 2.5^64^. Logarithm of the odds (LOD) scores were calculated for all pairs of loci. The order of loci with LOD scores ≥3 was determined based on multipoint linkage analysis. Loci with two-point LOD scores ≥4 that were not mapped with the multipoint analysis were additionally fitted. Genetic maps for the JF, WE, and JO lineages were generated with the multifamily datasets. To assess the patterns of recombination suppression in the maternal and paternal meioses of the hybrid crosses, comparative mapping analyses were performed based on female and male maps. Given that informative polymorphic loci could vary among the mapping crosses, comparative analyses were conducted with the aid of a re-assembled *G. aculeatus* genome sequence^65^, which is a modification of the original genome sequence^66^.

### QTL mapping

To investigate whether LG12 is responsible for sex determination in the JF, WE, and JO lineages, association and mapping analyses were conducted on the mapping families of these lineages, as well as on other crosses, with the 60 STR loci located on *G. aculeatus* Chr12 and Chr7. First, associations of phenotypic sex with maternally and paternally segregating alleles were examined separately to identify the sex-determining region and heterogametic sex. The significance of association was assessed based on Fisher’s exact test with Bonferroni correction. Second, QTL mapping was performed based on the Haseman–Elston regression method^67^ with the Visscher-Hopper correction^68^ using GridQTL^69^. Significant QTL were identified using the sib-pair model based on the 5% experiment-wide threshold of *F*-statistics with 10,000 permutations. The 95% confidence intervals of the QTL locations were estimated with 10,000 bootstrap replicates. Genetic maps of the heterogametic sex were used for the mapping analyses. In the crosses where no association with sex was identified, the sex-averaged maps were used. Phenotypic sex was identified based on visual inspection of gonads. For the JO lineage, only the two pure families were used for the association and QTL analyses. The numbers of females and males were, respectively, 70 and 72 in the JF families, 72 and 72 in the WE families, 29 and 26 in the JO families, 111 and 105 in the backcross between the JF and WE lineages, 70 and 74 in the F_2_ cross between a WE female and an EE male, and 67 and 72 in the F_2_ cross between an EE female and a WE male. The association analysis was also conducted for the F_1_ hybrids between a JO female and a JF male (13 females and 21 males). In addition, the association analysis was performed in a cross between a heterogametic female (ZW) and a heterogametic male (XY) to investigate whether the sex of their hybrids is determined by the ZW or XY system. F_1_ hybrids between a JF-YON female and a JB-BIW male were produced by artificial fertilization with F_1_ progeny of these populations and reared under the same conditions as those used for the mapping families of the JF and other lineages. The association analysis in this cross was conducted with 16 females and 16 males based on 13 polymorphic loci distributed across LG12.

Mapping analyses were also performed for male and female fertility in the individuals of the backcross between the JF and WE lineages. These individuals were produced when the parental fish were at 34 to 43 weeks post-hatching. The fertility of the backcross individuals was evaluated at 40 weeks post-hatching when most males showed nuptial coloration and many females were gravid. The gonad index and fertility of males were examined as described above. Out of the 105 males, 90 mature individuals with nuptial coloration were used for the mapping analyses. The gonad index of males in the backcross progeny was similar to that in the JF lineage (Mann–Whitney *U* test, *P* > 0.05). Fertile and sterile individuals were classified based on the presence and absence of sperm, respectively. In addition, as an index of female fertility, the gravidity status of females was determined based on the presence or absence of mature eggs that could be squeezed out. The effects of tank, clutch, and body weight were not considered in the analyses since there was no association between these factors and the fertility of males (chi-square test for tank and clutch, Mann–Whitney *U* test for body weight, *P* > 0.05) or the gravidity status of females (chi-square test for tank and clutch, Mann–Whitney U test for body weight, *P* > 0.05). The mapping analyses were conducted using the sex-averaged genetic map under the same conditions and criteria as those used for sex. In addition to the mapping analyses, the proportions of fertile males and gravid females were examined in individuals with each genotypic combination of grandparental JF and WE alleles at loci across LG12.

## Supplementary information

Supplementary Information

Description of Additional Supplementary Files

Supplementary Data 1

Supplementary Data 2

## Data Availability

Genotype data that support the findings of this study are available from Dryad under accession number 10.5061/dryad.3df3059. All mitochondrial sequence data used in this study are available from GenBank under accession numbers KR779233–KR779244 and KY819041–KY819043. All other data that support the findings of this study are available from the corresponding author on reasonable request.
