# Supplementary material for: The evolution of sex determination associated with a chromosomal inversion
Source: Nat Commun. 2019 Jan 11;10:145. doi: 10.1038/s41467-018-08014-y (PMC6329827; doi:10.1038/s41467-018-08014-y)
Supplement: Supplementary file 1 — Supplementary Information [file 41467_2018_8014_MOESM1_ESM.pdf]

Supplementary Information

The evolution of sex determination associated with a chromosomal inversion

Natri *et al.*

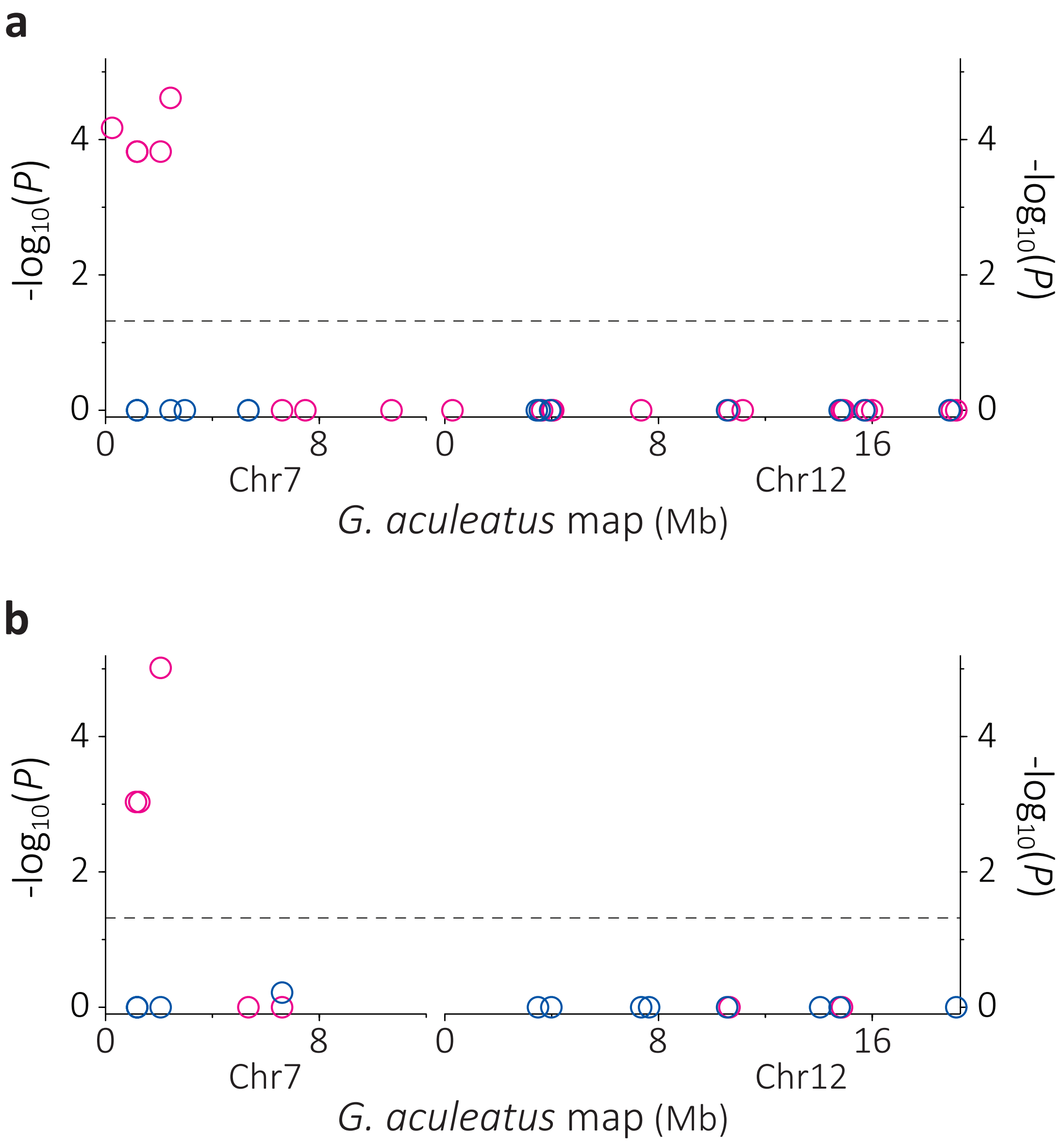

Supplementary Figure 1 | Association between sex and loci in the region corresponding to LG12. (**a**) F_1 hybrids between a JO female and a JF male. (**b**) F_1 hybrids between a JF female and a JB male. Maternally and paternally segregating alleles are indicated in red and blue, respectively.

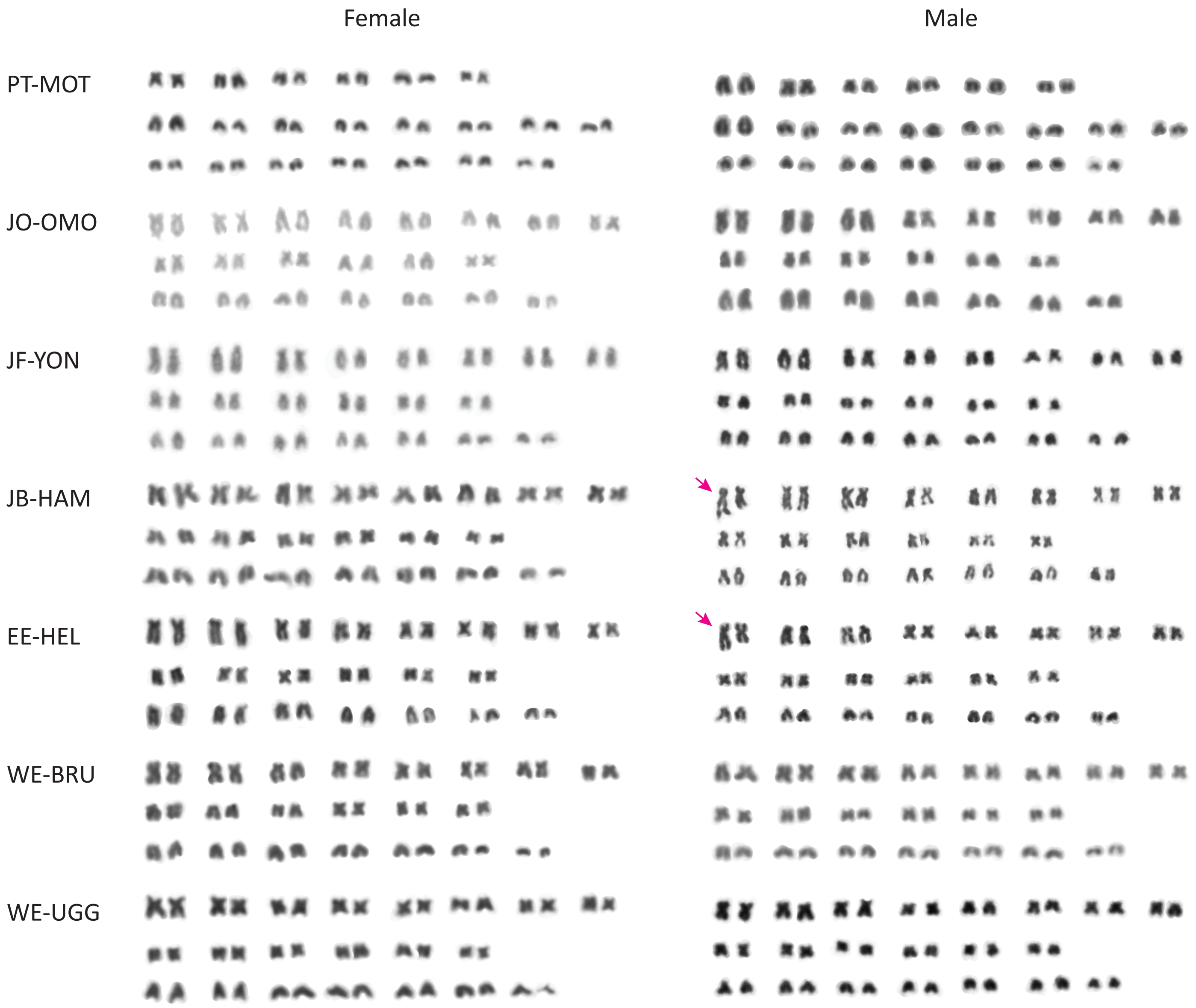

Supplementary Figure 2 | Karyotypes in *Pungitius* populations. Karyotypes of females (left) and males (right) in seven populations from six *Pungitius* lineages (PT, JO, JF, JB, EE and WE) are shown. Red arrows indicate large, male-specific chromosomes.

Supplementary Table 1 | Lineages and sampling sites of 29 *Pungitius* populations used in this study.

Lineage	Population ID	Sampling site	Coordinates
P. tymensis	PT-MOT	Motosakimui River, Shibetsu, Japan	43° 50' N, 145° 05' E
P. tymensis	PT-OBO	Oboro River basin, Akkeshi, Japan	43° 02' N, 144° 44' E
Japanese Omono	JO-OMO	Omono River basin, Daisen, Japan	39° 25' N, 140° 33' E
Japanese freshwater	JF-SHI	Shibetsu River, Nakashibetsu, Japan	43° 37' N, 145° 04' E
Japanese freshwater	JF-SOM	Shiomi River, Akkeshi, Japan	43° 02' N, 144° 51' E
Japanese freshwater	JF-OBO	Oboro River basin, Akkeshi, Japan	43° 02' N, 144° 44' E
Japanese freshwater	JF-KIE	Kievka River, Kievka, Russia	42° 53' N, 133° 39' E
Japanese freshwater	JF-YON	Yoneshiro River, Odate, Japan	40° 16' N, 140° 29' E
Japanese freshwater	JF-TAI	Tainai River basin, Tainai, Japan	38° 06' N, 139° 24' E
Japanese brackish-water	JB-BLS	Bolshaya River, Kamchatka, Russia	52° 45' N, 156° 15' E
Japanese brackish-water	JB-HAM	Hamanaka Bay, Hamanaka, Japan	43° 05' N, 145° 07' E
Japanese brackish-water	JB-SOM	Shiomi River, Akkeshi, Japan	43° 02' N, 144° 51' E
Japanese brackish-water	JB-WAT	Watari-Chirippu pond, Hamanaka, Japan	43° 02' N, 145° 03' E
Eastern North American	EA-BAF	Baffin Island, Canada	62° 52' N, 67° 21' W
Central North American	CA-FLO	Floatingstone Lake, Alberta, Canada	54° 14' N, 111° 38' W
Eastern European	EE-L1	Lake 1, Norrbotten, Sweden	67° 54' N, 20° 51' E
Eastern European	EE-LEV	White Sea, Levin Navolok, Russia	66° 17' N, 33° 26' E
Eastern European	EE-PYO	Pyöreälampi, Kuusamo, Finland	66° 16' N, 29° 26' E
Eastern European	EE-ONE	Lake Onega, Karelia, Russia	61° 35' N, 34° 38' E
Eastern European	EE-SAI	Lake Saimaa, Taipalsaari, Finland	61° 29' N, 27°29' E
Eastern European	EE-HEL	Baltic Sea, Helsinki, Finland	60° 12' N, 25° 11' E
Western European	WE-UGG	Uggdalsvatnet, Norway	63° 57' N, 10° 25' E
Western European	WE-ENG	Engervann, Bærum, Norway	59° 54' N, 10° 32' E
Western European	WE-GUD	Guden, Silkeborg, Denmark	56° 11' N, 9° 38' E
Western European	WE-HAR	Harelaw dam, Scotland, United Kingdom	55° 45' N, 4° 25' W
Western European	WE-NOR	North Sea, Emmerlev Klev, Denmark	54° 59' N, 8° 40' E
Western European	WE-BRU	Brugse polders, Maldegem, Belgium	51° 10' N, 3° 28' E
Western European	WE-MON	Montagny-lès-Seurre, France	47° 01' N, 5° 15' E
P. laevis	PL-LAR	Lary River, France	45° 14' N, 0° 13' W

Supplementary Table 2 | Genetic variation at 46 autosomal loci, male-specific alleles at six *G. aculeatus* Chr12 loci and cytochrome *b* haplotypes in 29 *Pungitius* populations.

Lineage	Population ID	STR					Cytochrome *b*	
		Sample *n*	Autosomal (46 loci)		*G. aculeatus* Chr12 (6 loci)		Sample *n*	Haplotype ID (*n*)
			P	H_E	*P*	Locus with male-specific allele (bp)		
P. tymensis	PT-MOT	36	35	0.345	3	-	3	Pun132 (3)
P. tymensis	PT-OBO	36	23	0.223	3	-	3	Pun133 (3)
Japanese Omono	JO-OMO	49	31	0.301	2	-	5	Pun110 (5)
Japanese freshwater	JF-SHI	48	39	0.385	4	-	5	J2 (5)
Japanese freshwater	JF-SOM	18	43	0.518	6	-	8	J1 (3), J2 (1), J4 (1), J5 (2), Pun100 (1)
Japanese freshwater	JF-OBO	48	42	0.521	5	-	6	J1 (1), J2 (1), J3 (1), J4 (1), J5 (2)
Japanese freshwater	JF-KIE	10	40	0.483	4	-	4	Pun135 (4)
Japanese freshwater	JF-YON	48	35	0.273	4	-	5	Pun101 (2), Pun102 (2), Pun103 (1)
Japanese freshwater	JF-TAI	48	42	0.516	4	-	5	Pun104 (3), Pun105 (1), Pun106 (1)
Japanese brackish-water	JB-BLS	11	44	0.706	6	Ppsm11 (189–197), Umf5 (179), Ppsm6 (232), Ppsm3 (182–186), Stn19 (180)	5	Pun136 (3), Pun137 (1), Pun138 (1)
Japanese brackish-water	JB-HAM	48	44	0.629	6	Ppsm11 (195), Umf5 (179), Ppsm6 (232), Ppsm5 (229), Stn19 (174)	6	J5 (1), Pun107 (3), Pun108 (1), Pun109 (1)
Japanese brackish-water	JB-SOM	20	44	0.630	6	Ppsm11 (195), Umf5 (179), Ppsm6 (232), Ppsm5 (229), Ppsm3 (194), Stn19 (174)	6	J5 (2), Pun107 (4)
Japanese brackish-water	JB-WAT	-	-	-	-	-	8	Pun107 (5), Pun147 (2), Pun148 (1)
Eastern North American	EA-BAF	23	20	0.140	6	Ppsm11 (189), Umf5 (179), Ppsm6 (232), Ppsm5 (229), Ppsm3 (182), Stn19 (180)	10	Pun111 (9), Pun112 (1)
Central North American	CA-FLO	36	36	0.199	5	Ppsm11 (193), Umf5 (179), Ppsm6 (232), Ppsm5 (217), Ppsm3 (178–209)	5	Pun139 (2), Pun140 (1), Pun141(1), Pun142 (1)
Eastern European	EE-L1	40	33	0.299	5	Ppsm11 (197), Ppsm6 (232), Ppsm5 (220), Ppsm3 (180), Stn19 (176)	3	E7 (2), E8 (1)
Eastern European	EE-LEV	40	43	0.532	6	Ppsm11 (197), Ppsm6 (232), Ppsm5 (229), Ppsm3 (184), Stn19 (176)	3	E3 (1), E11 (1), E12 (1)
Eastern European	EE-PYO	-	-	-	-	-	4	E3 (4)
Eastern European	EE-ONE	8	44	0.522	5	Ppsm11 (197), Ppsm6 (232), Ppsm5 (220), Ppsm3 (188), Stn19 (176)	3	E3 (2), E20 (1)
Eastern European	EE-SAI	40	45	0.518	5	Ppsm11 (197), Ppsm6 (232), Ppsm3 (180), Stn19 (176–180)	4	E3 (2), E21 (1), E22 (1)
Eastern European	EE-HEL	40	45	0.560	5	Ppsm11 (197), Ppsm6 (232), Ppsm5 (229), Ppsm3 (168–172), Stn19 (176)	4	E3 (4)
Western European	WE-UGG	-	-	-	-	-	8	Pun124 (8)
Western European	WE-ENG	41	46	0.577	5	-	6	E28 (3), E29 (1), E30 (2)
Western European	WE-GUD	32	39	0.390	5	Ppsm11 (197), Ppsm6 (232), Ppsm5 (229), Ppsm3 (188), Stn19 (176)	3	E31 (2), E33 (1)
Western European	WE-HAR	10	38	0.422	3	-	3	E34 (3)
Western European	WE-NOR	-	-	-	-	-	6	Pun143 (4), Pun149 (2)
Western European	WE-BRU	24	44	0.489	5	-	5	Pun143 (1), Pun144 (2), Pun145 (1), Pun146 (1)
Western European	WE-MON	8	40	0.414	5	-	7	E37 (3), E38 (4)
P. laevis	PL-LAR	10	32	0.410	4	-	6	Pun120 (2), Pun122 (3), Pun123 (1)
Gasterosteus aculeatus	*G. aculeatus*	24	44	0.644	6	-	1	Gac1 (1)

P, the number of polymorphic loci; H_E, mean expected heterozygosity.

Supplementary Table 3 | Average genetic distances (D_A) between PT, JO, JF and WE lineages and X and Y haplotypes of XY lineages based on 35 *G. aculeatus* Chr12 loci.

	PT lineage	JO lineage	JF lineage	WE lineage	X haplotype	Y haplotype
PT lineage	-	-	-	-	-	-
JO lineage	0.794 (1)	-	-	-	-	-
JF lineage	0.849 [0.750–0.948] (3)	0.870 [0.825–0.915] (3)	0.489 [0.409–0.568] (3)	-	-	-
WE lineage	0.926 [0.878–0.974] (3)	0.906 [0.886–0.926] (3)	0.878 [0.860–0.896] (9)	0.312 [-0.025–0.650] (3)	-	-
X haplotype	0.909 [0.864–0.954] (5)	0.919 [0.884–0.955] (5)	0.898 [0.882–0.914] (15)	0.593 [0.525–0.660] (15)	0.612 [0.454–0.771] (10)	-
Y haplotype	0.899 [0.868–0.929] (5)	0.930 [0.895–0.965] (5)	0.621 [0.588–0.654] (15)	0.918 [0.902–0.934] (15)	0.918 [0.901–0.934] (25)	0.564 [0.391–0.736] (10)

The number of population pairs is shown in parentheses. Numbers in brackets indicate 95% confidence intervals.

Supplementary Table 4 | Egg number, fertilization rate, hatching rate and larval survival in five *Pungitius* lineages and their crosses.

Cross		Offspring	Cross *n*	*n* of families used†		Egg *n*	Fertilization (%)	Hatching (%)	Larval survival (%)
Female	Male			Female	Male	(mean [range])	(mean ± s.e.m.)	(mean ± s.e.m.)	(mean ± s.e.m.)
Population									
JO-OMO	JO-OMO	JO-OMO	3	3	3	61.3 [44–75] (3)	100.0 ± 0.0 (3)	62.9 ± 15.6 (3)	92.9 ± 7.1 (3)
JF-YON	JF-YON	JF-YON	15	9	7	37.8 [15–87] (15)	98.3 ± 1.2 (15)	88.2 ± 2.2 (15)	98.5 ± 0.5 (15)
JB-HAM	JB-HAM	JB-HAM	15	8	11	38.1 [16–75] (15)	99.3 ± 0.5 (15)	92.3 ± 2.1 (15)	99.5 ± 0.4 (15)
EE-HEL	EE-HEL	EE-HEL	15	15	11	50.3 [17–99] (15)	98.6 ± 1.3 (15)	88.3 ± 2.9 (15)	97.3 ± 1.1 (15)
WE-BRU	WE-BRU	WE-BRU	15	10	10	65.1 [25–126] (15)	99.5 ± 0.3 (15)	92.8 ± 1.4 (15)	98.6 ± 0.6 (15)
Interlineage cross									
JO-OMO	JF-YON	F_1(JO × JF)	3	3	3	44.0 [40–52] (3)	100.0 ± 0.0 (3)	78.4 ± 9.6 (3)	98.4 ± 1.6 (3)
JF-YON	JO-OMO	F_1(JF × JO)	6	5	6	67.8 [33–86] (6)	98.9 ± 0.6 (6)	75.9 ± 5.4 (6)	96.2 ± 1.9 (6)
JO-OMO	JB-HAM	F_1(JO × JB)	4	4	4	47.5 [32–59] (4)	90.4 ± 6.0 (4)	87.3 ± 4.5 (4)	99.5 ± 0.5 (4)
JB-HAM	JO-OMO	F_1(JB × JO)	6	5	6	58.0 [45–77] (6)	99.7 ± 0.3 (6)	89.2 ± 3.8 (6)	97.8 ± 0.8 (6)
JO-OMO	EE-HEL	F_1(JO × EE)	4	4	4	40.3 [35–50] (4)	99.0 ± 1.0 (4)	77.1 ± 11.3 (4)	86.1 ± 7.3 (4)
EE-HEL	JO-OMO	F_1(EE × JO)	6	6	6	70.0 [40–158] (6)	100.0 ± 0.0 (6)	87.6 ± 2.2 (6)	97.8 ± 1.0 (6)
JO-OMO	WE-BRU	F_1(JO × WE)	3	3	3	53.3 [40–65] (3)	100.0 ± 0.0 (3)	66.8 ± 21.9 (3)	94.6 ± 4.0 (3)
WE-BRU	JO-OMO	F_1(WE × JO)	6	6	6	61.8 [50–77] (6)	92.7 ± 6.4 (6)	88.1 ± 4.4 (6)	98.6 ± 1.2 (6)
JF-YON	JB-HAM	F_1(JF × JB)	3	3	3	50.3 [42–61] (3)	100.0 ± 0.0 (3)	84.6 ± 3.8 (3)	98.2 ± 0.9 (3)
JB-HAM	JF-YON	F_1(JB × JF)	6	4	3	43.2 [32–61] (6)	99.1 ± 0.9 (6)	88.8 ± 4.6 (6)	99.6 ± 0.4 (6)
JF-YON	EE-HEL	F_1(JF × EE)	4	3	4	45.5 [26–65] (4)	99.0 ± 1.1 (4)	92.0 ± 2.7 (4)	99.6 ± 0.4 (4)
EE-HEL	JF-YON	F_1(EE × JF)	4	4	3	55.0 [34–70] (4)	95.6 ± 4.4 (4)	95.7 ± 1.6 (4)	98.7 ± 0.4 (4)
JF-YON	WE-BRU	F_1(JF × WE)	3	3	3	38.3 [28–44] (3)	98.4 ± 1.6 (3)	90.3 ± 4.4 (3)	100.0 ± 0.0 (3)
WE-BRU	JF-YON	F_1(WE × JF)	5	4	3	55.8 [50–71] (5)	100.0 ± 0.0 (5)	93.5 ± 2.7 (5)	96.5 ± 3.1 (5)
JB-HAM	EE-HEL	F_1(JB × EE)	6	5	4	47.8 [34–67] (6)	100.0 ± 0.0 (6)	89.9 ± 3.4 (6)	99.0 ± 1.0 (6)
EE-HEL	JB-HAM	F_1(EE × JB)	5	5	5	56.2 [39–78] (5)	100.0 ± 0.0 (5)	95.3 ± 1.6 (5)	97.9 ± 1.1 (5)
JB-HAM	WE-BRU	F_1(JB × WE)	7	5	5	40.4 [19–49] (7)	99.6 ± 0.4 (7)	89.9 ± 3.6 (7)	98.6 ± 1.0 (7)
WE-BRU	JB-HAM	F_1(WE × JB)	7	5	5	54.9 [37–73] (7)	100.0 ± 0.0 (7)	92.0 ± 2.5 (7)	98.6 ± 0.5 (7)
EE-HEL	WE-BRU	F_1(EE × WE)	10	10	10	59.1 [20–98] (10)	99.8 ± 0.2 (10)	88.2 ± 4.5 (10)	97.6 ± 1.1 (10)
WE-BRU	EE-HEL	F_1(WE × EE)	10	10	10	89.4 [31–132] (10)	99.6 ± 0.3 (10)	94.4 ± 2.3 (10)	98.5 ± 0.8 (10)

The number of families is shown in parentheses. †Wild-caught individuals are counted as different families.
Hatching and larval survival rates are represented based on fertilized and hatched eggs, respectively.

Supplementary Table 5 | Survival and sex ratio in five *Pungitius* lineages and their hybrids.

Cross		Offspring	Cross *n*	*n* of families used†		Egg *n*	Fertilized egg *n*	Hatched egg *n*	Larval survival *n*	Adult *n*	Adult female *n*	Adult male *n*
Female	Male			Female	Male			(/fertilized egg)	(/fertilized egg)	(/fertilized egg)	(/adult)	(/adult)
Population												
JO-OMO	JO-OMO	JO-OMO	2	2	2	140	140	110 (78.6%)	110 (78.6%)	57 (40.7%)	30 (52.6%)	27 (47.4%)
JF-YON	JF-YON	JF-YON	12	7	7	466	446	385 (86.3%)	379 (85.0%)	243 (54.5%)	119 (49.0%)	124 (51.0%)
JB-HAM	JB-HAM	JB-HAM	15	8	11	572	567	523 (92.2%)	520 (91.7%)	391 (69.0%)	190 (48.6%)	201 (51.4%)
EE-HEL	EE-HEL	EE-HEL	12	12	10	517	508	436 (85.8%)	424 (83.5%)	244 (48.0%)	132 (54.1%)	113 (46.3%)
WE-BRU	WE-BRU	WE-BRU	12	9	9	815	812	761 (93.7%)	748 (92.1%)	351 (43.2%)	176 (50.1%)	175 (49.9%)
Interlineage F_1 hybrid												
JO-OMO	JF-YON	F_1(JO × JF)	3	3	3	132	132	104 (78.8%)	102 (77.3%)	84 (63.6%)	35 (41.7%)	49 (58.3%)
JF-YON	JO-OMO	F_1(JF × JO)	6	5	6	407	402	305 (75.9%)	296 (73.6%)	180 (44.8%)	77 (42.8%)	103 (57.2%)
JO-OMO	JB-HAM	F_1(JO × JB)	4	4	4	190	172	153 (89.0%)	152 (88.4%)	111 (64.5%)	49 (44.1%)	62 (55.9%)
JB-HAM	JO-OMO	F_1(JB × JO)	6	5	6	348	347	310 (89.3%)	304 (87.6%)	155 (44.7%)	36 (23.2%)***	119 (76.8%)***
JO-OMO	EE-HEL	F_1(JO × EE)	3	3	3	124	122	85 (69.7%)	71 (58.2%)	49 (40.2%)	23 (46.9%)	26 (53.1%)
EE-HEL	JO-OMO	F_1(EE × JO)	4	4	4	328	328	291 (88.7%)	287 (87.5%)	81 (24.7%)	29 (35.8%)	52 (64.2%)
JO-OMO	WE-BRU	F_1(JO × WE)	3	3	3	160	160	99 (61.9%)	96 (60.0%)	84 (52.5%)	33 (39.3%)	51 (60.7%)
WE-BRU	JO-OMO	F_1(WE × JO)	5	5	5	308	275	245 (89.1%)	244 (88.7%)	102 (37.1%)	17 (16.7%)***	85 (83.3%)***
JF-YON	JB-HAM	F_1(JF × JB)	3	3	3	151	151	128 (84.8%)	126 (83.4%)	86 (57.0%)	41 (47.7%)	45 (52.3%)
JB-HAM	JF-YON	F_1(JB × JF)	6	4	3	259	257	231 (89.9%)	230 (89.5%)	179 (69.6%)	16 (8.9%)***	163 (91.1%)***
JF-YON	EE-HEL	F_1(JF × EE)	4	3	4	182	180	167 (92.8%)	166 (92.2%)	91 (50.6%)	53 (58.2%)	38 (41.8%)
EE-HEL	JF-YON	F_1(EE × JF)	4	4	3	220	214	205 (95.8%)	202 (94.4%)	100 (46.7%)	17 (17.0%)***	83 (83.0%)***
JF-YON	WE-BRU	F_1(JF × WE)	3	3	3	115	113	101 (89.4%)	101 (89.4%)	59 (52.2%)	24 (40.7%)	35 (59.3%)
WE-BRU	JF-YON	F_1(WE × JF)	5	4	3	279	279	261 (93.5%)	253 (90.7%)	126 (45.2%)	0 (0.0%)***	126 (100.0%)***
JB-HAM	EE-HEL	F_1(JB × EE)	6	5	4	287	287	258 (89.9%)	256 (89.2%)	178 (62.0%)	88 (49.4%)	90 (50.6%)
EE-HEL	JB-HAM	F_1(EE × JB)	5	5	5	281	281	269 (95.7%)	264 (94.0%)	129 (45.9%)	61 (47.3%)	68 (52.7%)
JB-HAM	WE-BRU	F_1(JB × WE)	7	5	5	283	282	259 (91.8%)	255 (90.4%)	166 (58.9%)	77 (46.4%)	89 (53.6%)
WE-BRU	JB-HAM	F_1(WE × JB)	7	5	5	384	384	357 (93.0%)	352 (91.7%)	238 (62.0%)	117 (49.2%)	121 (50.8%)
EE-HEL	WE-BRU	F_1(EE × WE)	3	3	3	125	125	92 (73.6%)	89 (71.2%)	64 (51.2%)	30 (46.9%)	34 (53.1%)
WE-BRU	EE-HEL	F_1(WE × EE)	4	4	4	426	426	376 (88.3%)	373 (87.6%)	134 (31.5%)	67 (50.0%)	67 (50.0%)

Significantly different from 1:1 at $P < 0.001$***. †Wild-caught individuals are counted as different families.

Supplementary Table 6 | Egg number, fertilization rate, hatching rate and larval survival in backcrosses between interlineage F_1 hybrids and parental populations.

Parent		Offspring	Cross *n*	*n* of families used†		Egg *n*	Fertilization (%)	Hatching (%)	Larval survival (%)
Female	Male			Female	Male	(mean [range])	(mean ± s.e.m.)	(mean ± s.e.m.)	(mean ± s.e.m.)
F_1(JO × JF)	JO-OMO	BC(F_1(JO×JF) × JO)	4	3	3	46.8 [28–83] (4)	93.3 ± 5.9 (4)	77.5 ± 14.1 (4)	100.0 ± 0.0 (4)
F_1(JO × JF)	JF-YON	BC(F_1(JO×JF) × JF)	13	3	5	51.0 [12–81] (13)	84.6 ± 5.5 (13)	84.4 ± 4.6 (13)	93.4 ± 3.6 (13)
JO-OMO	F_1(JO × JF)	BC(JO × F_1(JO×JF))	3	3	3	26.7 [18–34] (3)	85.5 ± 2.9 (3)	73.6 ± 11.1 (3)	100.0 ± 0.0 (3)
JF-YON	F_1(JO × JF)	BC(JF × F_1(JO×JF))	8	4	3	45.0 [32–66] (8)	99.1 ± 0.4 (8)	77.2 ± 5.5 (8)	97.2 ± 1.9 (8)
F_1(JF × JO)	JO-OMO	BC(F_1(JF×JO) × JO)	7	4	4	46.7 [27–99] (7)	88.6 ± 6.1 (7)	75.1 ± 2.9 (7)	99.2 ± 0.5 (7)
F_1(JF × JO)	JF-YON	BC(F_1(JF×JO) × JF)	14	4	7	50.7 [27–71] (14)	95.3 ± 2.6 (14)	82.6 ± 2.8 (14)	94.3 ± 2.4 (14)
JO-OMO	F_1(JF × JO)	BC(JO × F_1(JF×JO))	2	2	2	25.5 [18–33] (2)	94.0 ± 6.1 (2)	93.8 ± 0.7 (2)	98.2 ± 1.9 (2)
JF-YON	F_1(JF × JO)	BC(JF × F_1(JF×JO))	14	4	5	47.9 [36–110] (14)	99.5 ± 0.3 (14)	80.9 ± 4.2 (14)	97.0 ± 1.9 (14)
F_1(JO × JB)	JO-OMO	BC(F_1(JO×JB) × JO)	2	2	2	28.0 [25–31] (2)	98.4 ± 1.6 (2)	90.0 ± 10.0 (2)	98.4 ± 1.7 (2)
F_1(JO × JB)	JB-HAM	BC(F_1(JO×JB) × JB)	4	3	4	50.8 [31–83] (4)	100.0 ± 0.0 (4)	81.4 ± 3.1 (4)	90.0 ± 8.7 (4)
JO-OMO	F_1(JO × JB)	BC(JO × F_1(JO×JB))	2	2	1	19.0 [17–21] (2)	0.0 ± 0.0 (2)	(0)	(0)
JB-HAM	F_1(JO × JB)	BC(JB × F_1(JO×JB))	16	6	4	45.0 [15–83] (16)	45.6 ± 12.0 (16)	46.1 ± 7.7 (8)	65.7 ± 7.4 (8)
F_1(JB × JO)	JO-OMO	BC(F_1(JB×JO) × JO)	2	2	2	29.5 [24–35] (2)	98.6 ± 1.5 (2)	76.4 ± 2.9 (2)	100.0 ± 0.0 (2)
F_1(JB × JO)	JB-HAM	BC(F_1(JB×JO) × JB)	5	3	3	36.4 [22–79] (5)	100.0 ± 0.0 (5)	88.8 ± 3.2 (5)	95.4 ± 2.3 (5)
JO-OMO	F_1(JB × JO)	BC(JO × F_1(JB×JO))	6	6	5	25.2 [19–34] (6)	0.0 ± 0.0 (6)	(0)	(0)
JB-HAM	F_1(JB × JO)	BC(JB × F_1(JB×JO))	15	6	6	44.0 [15–112] (15)	4.3 ± 4.3 (15)	10.7 (1)	100 (1)
F_1(JO × EE)	JO-OMO	BC(F_1(JO×EE) × JO)	1	1	1	35.0 [35–35] (1)	100.0 (1)	31.4 (1)	90.9 (1)
F_1(JO × EE)	EE-HEL	BC(F_1(JO×EE) × EE)	4	2	4	77.3 [27–132] (4)	95.6 ± 3.0 (4)	55.2 ± 12.1 (4)	93.5 ± 1.5 (4)
JO-OMO	F_1(JO × EE)	BC(JO × F_1(JO×EE))	3	3	2	22.7 [10–35] (3)	0.0 ± 0.0 (3)	(0)	(0)
EE-HEL	F_1(JO × EE)	BC(EE × F_1(JO×EE))	9	5	4	44.8 [27–69] (9)	29.8 ± 15.2 (9)	50.5 ± 11.6 (3)	94.4 ± 5.6 (3)
F_1(EE × JO)	JO-OMO	BC(F_1(EE×JO) × JO)	3	3	3	41.3 [20–58] (3)	94.6 ± 0.8 (3)	52.0 ± 9.5 (3)	93.8 ± 2.0 (3)
F_1(EE × JO)	EE-HEL	BC(F_1(EE×JO) × EE)	7	3	6	33.3 [12–49] (7)	94.2 ± 2.4 (7)	63.0 ± 9.0 (7)	97.7 ± 1.3 (7)
JO-OMO	F_1(EE × JO)	BC(JO × F_1(EE×JO))	4	4	3	18.0 [13–24] (4)	0.0 ± 0.0 (4)	(0)	(0)
EE-HEL	F_1(EE × JO)	BC(EE × F_1(EE×JO))	15	4	7	43.5 [21–92] (15)	0.0 ± 0.0 (15)	(0)	(0)
F_1(JO × WE)	JO-OMO	BC(F_1(JO×WE) × JO)	3	1	2	24.7 [18–32] (3)	92.6 ± 7.4 (3)	61.8 ± 8.9 (3)	96.3 ± 3.7 (3)
F_1(JO × WE)	WE-BRU	BC(F_1(JO×WE) × WE)	8	2	5	45.9 [18–72] (8)	97.9 ± 1.7 (8)	76.9 ± 6.3 (8)	95.6 ± 1.6 (8)
JO-OMO	F_1(JO × WE)	BC(JO × F_1(JO×WE))	5	5	2	29.6 [12–69] (5)	26.0 ± 17.6 (5)	37.2 ± 2.8 (2)	83.4 ± 16.7 (2)
WE-BRU	F_1(JO × WE)	BC(WE × F_1(JO×WE))	13	6	3	62.0 [33–101] (13)	58.9 ± 12.1 (13)	66.7 ± 6.3 (9)	78.8 ± 7.1 (9)
F_1(WE × JO)	JO-OMO	BC(F_1(WE×JO) × JO)	7	4	5	35.3 [21–57] (7)	93.5 ± 4.8 (7)	70.4 ± 8.9 (7)	93.9 ± 1.8 (7)
F_1(WE × JO)	WE-BRU	BC(F_1(WE×JO) × WE)	11	5	6	52.7 [18–100] (11)	97.0 ± 2.2 (11)	78.9 ± 4.9 (11)	93.8 ± 2.2 (11)
JO-OMO	F_1(WE × JO)	BC(JO × F_1(WE×JO))	5	5	4	28.8 [13–59] (5)	0.0 ± 0.0 (5)	(0)	(0)
WE-BRU	F_1(WE × JO)	BC(WE × F_1(WE×JO))	16	4	5	61.0 [28–113] (16)	42.6 ± 10.7 (16)	67.1 ± 6.1 (9)	77.6 ± 7.3 (9)
F_1(JF × JB)	JF-YON	BC(F_1(JF×JB) × JF)	8	3	6	42.3 [33–59] (8)	99.5 ± 0.4 (8)	87.9 ± 4.7 (8)	89.3 ± 5.5 (8)
F_1(JF × JB)	JB-HAM	BC(F_1(JF×JB) × JB)	7	3	5	42.0 [27–55] (7)	100.0 ± 0.0 (7)	86.5 ± 4.9 (7)	91.4 ± 2.9 (7)
JF-YON	F_1(JF × JB)	BC(JF × F_1(JF×JB))	6	4	3	47.0 [19–72] (6)	0.0 ± 0.0 (6)**	(0)	(0)
JB-HAM	F_1(JF × JB)	BC(JB × F_1(JF×JB))	6	5	3	37.5 [23–58] (6)	0.0 ± 0.0 (6)**	(0)	(0)
F_1(JB × JF)	JF-YON	BC(F_1(JB×JF) × JF)	2	1	2	41.5 [41–42] (2)	100.0 ± 0.0 (2)	86.8 ± 1.3 (2)	94.5 ± 0.1 (2)
F_1(JB × JF)	JB-HAM	BC(F_1(JB×JF) × JB)	2	1	2	41.0 [37–45] (2)	100.0 ± 0.0 (2)	91.8 ± 2.8 (2)	96.3 ± 3.8 (2)
JF-YON	F_1(JB × JF)	BC(JF × F_1(JB×JF))	12	5	6	47.3 [28–68] (12)	0.0 ± 0.0 (12)***	(0)	(0)
JB-HAM	F_1(JB × JF)	BC(JB × F_1(JB×JF))	12	7	6	42.1 [16–78] (12)	0.0 ± 0.0 (12)***	(0)	(0)
F_1(JF × EE)	JF-YON	BC(F_1(JF×EE) × JF)	6	3	3	51.0 [29–70] (6)	99.8 ± 0.2 (6)	83.2 ± 3.7 (6)	94.2 ± 2.0 (6)
F_1(JF × EE)	EE-HEL	BC(F_1(JF×EE) × EE)	6	3	3	44.8 [31–60] (6)	98.9 ± 0.8 (6)	90.5 ± 4.4 (6)	86.6 ± 5.3 (6)
JF-YON	F_1(JF × EE)	BC(JF × F_1(JF×EE))	8	6	4	50.3 [37–83] (8)	0.0 ± 0.0 (8)***	(0)	(0)
EE-HEL	F_1(JF × EE)	BC(EE × F_1(JF×EE))	8	6	4	55.4 [23–89] (8)	0.0 ± 0.0 (8)***	(0)	(0)

JF-YON	F_1(EE × JF)	BC(JF × F_1(EE×JF))	8	6	4	47.1 [33–87] (8)	0.0 ± 0.0 (8)***	(0)	(0)
EE-HEL	F_1(EE × JF)	BC(EE × F_1(EE×JF))	8	6	4	50.1 [22–92] (8)	0.0 ± 0.0 (8)***	(0)	(0)
F_1(JF × WE)	JF-YON	BC(F_1(JF×WE) × JF)	7	3	3	57.7 [36–111] (7)	98.6 ± 1.3 (7)	92.3 ± 2.0 (7)	95.9 ± 1.3 (7)
F_1(JF × WE)	WE-BRU	BC(F_1(JF×WE) × WE)	7	3	5	54.6 [25–112] (7)	94.4 ± 5.1 (7)	89.1 ± 5.5 (7)	95.0 ± 1.1 (7)
JF-YON	F_1(JF × WE)	BC(JF × F_1(JF×WE))	8	4	4	44.6 [33–54] (8)	0.0 ± 0.0 (8)***	(0)	(0)
WE-BRU	F_1(JF × WE)	BC(WE × F_1(JF×WE))	8	5	4	71.9 [19–104] (8)	0.0 ± 0.0 (8)***	(0)	(0)
JF-YON	F_1(WE × JF)	BC(JF × F_1(WE×JF))	11	6	6	51.5 [33–96] (11)	0.0 ± 0.0 (11)***	(0)	(0)
WE-BRU	F_1(WE × JF)	BC(WE × F_1(WE×JF))	11	7	6	73.6 [18–109] (11)	0.0 ± 0.0 (11)***	(0)	(0)
F_1(JB × EE)	JB-HAM	BC(F_1(JB×EE) × JB)	12	6	7	47.8 [23–81] (12)	97.7 ± 2.3 (12)	89.7 ± 1.8 (12)	96.1 ± 2.8 (12)
F_1(JB × EE)	EE-HEL	BC(F_1(JB×EE) × EE)	12	6	6	43.8 [20–88] (12)	99.1 ± 0.6 (12)	92.6 ± 1.4 (12)	99.0 ± 0.7 (12)
JB-HAM	F_1(JB × EE)	BC(JB × F_1(JB×EE))	10	6	6	57.4 [32–101] (10)	98.0 ± 1.4 (10)	90.3 ± 3.3 (10)	98.5 ± 0.7 (10)
EE-HEL	F_1(JB × EE)	BC(EE × F_1(JB×EE))	10	6	6	63.7 [35–93] (10)	99.5 ± 0.5 (10)	91.9 ± 2.6 (10)	98.9 ± 0.5 (10)
F_1(EE × JB)	JB-HAM	BC(F_1(EE×JB) × JB)	10	7	7	41.4 [29–62] (10)	98.6 ± 1.4 (10)	92.6 ± 1.5 (10)	99.3 ± 0.4 (10)
F_1(EE × JB)	EE-HEL	BC(F_1(EE×JB) × EE)	10	6	7	39.8 [25–66] (10)	100.0 ± 0.0 (10)	91.3 ± 1.9 (10)	96.4 ± 1.0 (10)
JB-HAM	F_1(EE × JB)	BC(JB × F_1(EE×JB))	11	8	6	51.5 [25–78] (11)	99.2 ± 0.4 (11)	95.2 ± 1.1 (11)	99.5 ± 0.3 (11)
EE-HEL	F_1(EE × JB)	BC(EE × F_1(EE×JB))	10	5	5	57.9 [37–90] (10)	99.8 ± 0.2 (10)	93.0 ± 2.4 (10)	99.8 ± 0.2 (10)
F_1(JB × WE)	JB-HAM	BC(F_1(JB×WE) × JB)	11	5	7	71.2 [51–97] (11)	99.3 ± 0.7 (11)	95.8 ± 1.3 (11)	98.7 ± 0.4 (11)
F_1(JB × WE)	WE-BRU	BC(F_1(JB×WE) × WE)	11	5	5	65.6 [46–97] (11)	100.0 ± 0.0 (11)	95.5 ± 1.2 (11)	98.8 ± 0.4 (11)
JB-HAM	F_1(JB × WE)	BC(JB × F_1(JB×WE))	10	6	5	70.3 [29–130] (10)	98.5 ± 0.8 (10)	91.4 ± 2.2 (10)	99.4 ± 0.3 (10)
WE-BRU	F_1(JB × WE)	BC(WE × F_1(JB×WE))	10	5	5	62.9 [47–79] (10)	98.1 ± 0.8 (10)	93.5 ± 1.7 (10)	97.8 ± 1.0 (10)
F_1(WE × JB)	JB-HAM	BC(F_1(WE×JB) × JB)	11	6	7	59.3 [39–112] (11)	99.6 ± 0.3 (11)	92.7 ± 1.9 (11)	98.6 ± 0.7 (11)
F_1(WE × JB)	WE-BRU	BC(F_1(WE×JB) × WE)	10	5	7	49.8 [30–62] (10)	99.8 ± 0.2 (10)	96.0 ± 1.4 (10)	97.4 ± 0.9 (10)
JB-HAM	F_1(WE × JB)	BC(JB × F_1(WE×JB))	11	7	5	57.6 [29–92] (11)	98.5 ± 0.9 (11)	91.2 ± 2.2 (11)	98.8 ± 0.4 (11)
WE-BRU	F_1(WE × JB)	BC(WE × F_1(WE×JB))	10	5	5	61.7 [35–83] (10)	99.7 ± 0.2 (10)	92.3 ± 2.6 (10)	99.2 ± 0.4 (10)
F_1(EE × WE)	EE-HEL	BC(F_1(EE×WE) × EE)	7	3	3	46.1 [27–66] (7)	100.0 ± 0.0 (7)	94.6 ± 1.7 (7)	94.4 ± 4.4 (7)
F_1(EE × WE)	WE-BRU	BC(F_1(EE×WE) × WE)	7	3	3	42.0 [17–62] (7)	100.0 ± 0.0 (7)	96.8 ± 1.2 (7)	92.5 ± 5.4 (7)
EE-HEL	F_1(EE × WE)	BC(EE × F_1(EE×WE))	7	3	4	35.6 [18–51] (7)	100.0 ± 0.0 (7)	88.7 ± 4.5 (7)	98.7 ± 0.6 (7)
WE-BRU	F_1(EE × WE)	BC(WE × F_1(EE×WE))	7	3	4	36.9 [30–49] (7)	99.6 ± 0.4 (7)	97.5 ± 0.8 (7)	95.5 ± 2.9 (7)
F_1(WE × EE)	EE-HEL	BC(F_1(WE×EE) × EE)	8	4	3	48.8 [27–73] (8)	100.0 ± 0.0 (8)	95.5 ± 2.2 (8)	99.0 ± 0.8 (8)
F_1(WE × EE)	WE-BRU	BC(F_1(WE×EE) × WE)	8	4	3	39.0 [16–70] (8)	100.0 ± 0.0 (8)	98.5 ± 0.7 (8)	99.7 ± 0.3 (8)
EE-HEL	F_1(WE × EE)	BC(EE × F_1(WE×EE))	7	3	4	39.0 [20–58] (7)	100.0 ± 0.0 (7)	87.8 ± 4.0 (7)	98.7 ± 1.3 (7)
WE-BRU	F_1(WE × EE)	BC(WE × F_1(WE×EE))	7	3	4	38.6 [21–58] (7)	100.0 ± 0.0 (7)	92.6 ± 2.2 (7)	97.9 ± 1.1 (7)

The number of families is shown in parentheses. Significantly different from both parental populations at $P < 0.01$** or $P < 0.001$***.

†Wild-caught individuals are counted as different families. Hatching and larval survival rates are represented based on fertilized and hatched eggs, respectively.
