# Supplementary material for: The evolution of sex determination associated with a chromosomal inversion
Source: Nat Commun. 2019 Jan 11;10:145. doi: 10.1038/s41467-018-08014-y (PMC6329827; doi:10.1038/s41467-018-08014-y)
Supplement: Supplementary file 2 — Description of Additional Supplementary Files [file 41467_2018_8014_MOESM2_ESM.pdf]

Description of Additional Supplementary Files

File Name: Supplementary Data 1
Description: STR markers used in this study and their locations in the G. aculeatus genome.

File Name: Supplementary Data 2
Description: Genetic variation and male-specific alleles at 35 *G. aculeatus* Chr12 loci in 13 *Pungitius* populations.
